# Impact framework: A python package for writing data analysis workflows to interpret microbial physiology

**DOI:** 10.1016/j.mec.2019.e00089

**Published:** 2019-04-04

**Authors:** Naveen Venayak, Kaushik Raj, Radhakrishnan Mahadevan

**Affiliations:** a Department of Chemical Engineering and Applied Chemistry, University of Toronto, 200 College Street, Toronto, ON, M5S 3E5, Canada; b Institute of Biomaterials and Biomedical Engineering, University of Toronto, 164 College Street, Toronto, ON, M5S 3G9, Canada

## Abstract

Microorganisms can be genetically engineered to solve a range of challenges in diverse including health, environmental protection and sustainability. The natural complexity of biological systems makes this an iterative cycle, perturbing metabolism and making stepwise progress toward a desired phenotype through four major stages: design, build, test, and data interpretation. This cycle has been accelerated by advances in molecular biology (e.g. robust DNA synthesis and assembly techniques), liquid handling automation and scale-down characterization platforms, generating large heterogeneous data sets. Here, we present an extensible Python package for scientists and engineers working with large biological data sets to interpret, model, and visualize data: the IMPACT (Integrated Microbial Physiology: Analysis, Characterization and Translation) framework. Impact aims to ease the development of Python-based data analysis workflows for a range of stakeholders in the bioengineering process, offering open-source tools for data analysis, physiology characterization and translation to visualization. Using this framework, biologists and engineers can opt for reproducible and extensible programmatic data analysis workflows, mediating a bottleneck limiting the throughput of microbial engineering. The Impact framework is available at https://github.com/lmse/impact.

## Highlights

• IMPACT - an open source Python-based framework for data analysis and visualization was developed.
• IMPACT was used to demonstrate visualization of microbial fermentation data.
• A carbon balance was calculated and metabolic flux was visualized on metabolic maps using data parsed by IMPACT.

## 1 Introduction

Microorganisms serve important roles in diverse areas of fundamental and applied research such as health and sustainability. Modern tools in biotechnology have accelerated the characterization and engineering of microbes to face these new challenges. In the past two decades, there have been significant advancements in the field of systems biology, to rapidly characterize and develop models for organisms of interest (Metzker, 2009; King et al., 2015b), and in the field of synthetic biology, to design and synthesize biological constructs (Hillson et al., 2012; Cameron et al., 2014). Simultaneously, laboratory throughput has been significantly increased, owing to advanced analytics and automation (Huber et al., 2009; Jacques et al., 2017). These advancements have drastically improved our understanding and ability to engineer biology to solve new challenges (Lee et al., 2012).

From cell culture experiments, a microbe's physiology and metabolic state can be assessed, often studied in batch, semi-batch, or chemostat culture. To engineer these microbes, their metabolism is perturbed based on metabolic hypotheses to be tested. This process is generally iterative, composed of four main stages: design, build, test, and learn (DBTL) (Liu et al., 2015). Although our understanding of microbes is rapidly improving, reaching desired microbial performance requires many cycles through this process. Metabolic engineering continues to strive for modular and predictable designs common to other engineering disciplines (Nielsen et al., 2016; Salis et al., 2009; Olson et al., 2014), but the complexity of metabolism imposes significant challenges.

To overcome this lack of predictability, large libraries of strains can be developed and characterized. To improve the throughput, fermentations have been scaled-down to a microtiter plate and even to the droplet scale (Wang et al., 2014), generating significant amounts of features (e.g. analytes) to understand the metabolism of the microorganism(s) involved. The complexity of acquiring key data types can vary significantly, especially at different fermentation scales; accordingly, the measured data types can vary significantly. At the bioreactor scale, on-line pH, dissolved oxygen, and feed additions are commonly monitored. Modern methods also exist to monitor these features at the microplate scale (Unthan et al., 2015), although they generally require specialized equipment. Typically, at any scale, the composition of the fermentation medium can be sampled (given sufficient volume) and analyzed by numerous chromatography methods, the gas phase can be sampled or monitored continuously using a process mass spectrometer and the biomass concentration can be monitored using optical density. Advancements in mass spectrometry have enabled the rapid generation of metabolomic profiles of strains with relatively small sample volumes (Petzold et al., 2015). This type of data can be synthesized into some key performance indicators, such as titer, rate, and yield (TRY) for measured components, all of which are important to understanding the physiology of the microbe.

Given the quantities of biological data being generated, there has been widespread standardization of data formats, databases, and analysis tools (NCBI Resource Coordinators, 2016; Galdzicki et al., 2014; King et al., 2016; Mahadevan et al., 2005). Biological systems are often characterized using -omics technologies, including genomics, proteomics, metabolomics and fluxomics (describing the flow of metabolites through metabolism). The fluxome is generally determined using cultures with isotopic labeling and mass spectrometry data to estimate fluxes using a least squared algorithm (Zamboni et al., 2009; Quek et al., 2009; Tang et al., 2009); however, this can require complex setups and expensive substrates. The predictive power of these constraint based metabolic models has been improving, and they can often be used to predict complex phenotypes (Bordbar et al., 2014). In lieu of the detail required to determine the fluxome, culture features such as changes in product titers and substrate concentrations can be used to estimate microbial exchange fluxes. Then, the model can be constrained using these rates, and an objective function can be used to predict an internal flux distribution. These types of algorithmic analyses can be challenging when structured data are lacking.

Typically, data are handled in spreadsheet applications using custom data processing templates. This process is cumbersome, non-transparent and it does not lend itself to facile data sharing. In the recent past, several software tools have been developed to serve as standardized repositories for experimental data storage (Rocca-Serra et al., 2010; Morrell et al., 2017). However, an open-source framework that facilitates storage and analysis of experimental data to gain useful insights from the data does not exist. Here we present Impact framework (integrated microbial physiology: analysis, characterization, translation framework), an integrated framework for analyzing microbial physiology. The Impact framework aims to aid scientists and engineers analyze, characterize and translate raw data describing microbial physiology. To do so, the Impact framework relies on a standard metadata schema to describe experiments and uses this data to parse it into a logical hierarchical format. From here, features are extracted to provide an augmented view of this data. Finally, this organized data structure can be queried for plotting, or downstream analyses in Python. Alternatively, these analyses can be perform in a variety of tools which can connect and utilize data from a relational database (e.g. Excel, Tableau).

The framework is modularly designed to promote contribution from a range of stakeholders in the bioengineering pipeline. To do so, the Impact framework relies on a number of open-source packages, to keep the code base small and agile. Thus, the framework is not aimed to be a “black-box” solution for data analysis, but rather promote contributions to the data warehousing and analysis pipeline in order to arrive at a community-driven consensus on data analysis best practices. Through standards in the software development life cycle (e.g. testing and continuous integration), we anticipate that contributions to the framework wcan remain comprehensive and robust.

## 2 Methods

### 2.1 Architecture

**Fig. 1:**
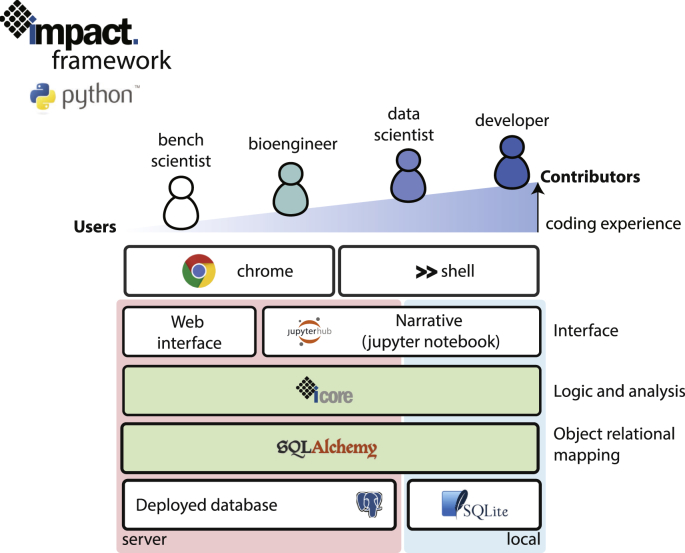
The software tool stack that depicts the tools available for various users to access and handle experimental data. Users with varying level of coding expertise can employ the Impact framework using different interfaces to read and analyze their data before storing it in a database.

The Impact framework aims to provide a high-level interface for analyzing data for microbial physiology. This process often involves a range of stakeholders with varying levels of coding experience, from biologists to software developers. The complex and iterative nature of biological engineering necessitates communication between many of these stakeholders, accelerated by easy access to underlying data sets. Impact can be used as a framework to power a range of workflows catering to unique needs in the process of engineering biological systems, ensuring a unified structure for all analysis pipelines. For example, the framework can be used to power a simple front-end web application, as well as complex programmatic data analysis pipelines. We expect this parallel structure to improve communication between stakeholders and reduce development times (Fig. 1).

This concept has motivated a majority of design decisions in the Impact framework. First, the most important design decision was the choice of programming language: Python. This choice was clear owing to the popularity of Python amongst developers and data scientists (https://insights.stackoverflow.com/survey/2018/#technology). In addition, a significant number of packages for scientific computing (e.g. numpy, scipy, and scikit-learn) and continuous integration (e.g. unittests, travis ci, and coverage) exist for Python.

Although this package can be used through any preferred interface, we encourage the use of Jupyter notebooks (https://jupyter.org), since it allows workflows to be represented as narratives, which serve as a unified place for the storage of the motivation, workflow, and results of a study. This drastically improves the ability of workflows to be shared and interpreted.

### 2.2 Data structure

#### 2.2.1 Models

Being written entirely in Python, the Impact framework is implemented using object oriented programming (OOP). Using inheritance, native data structures can be extended to include new analytes, features, or analysis methodologies. Furthermore, this structure allows the convenient application of an object-relational mapping (ORM) which can translate these Python data structures into a range of relational databases for facile storing and query.

#### 2.2.2 Schema

**Fig. 2:**
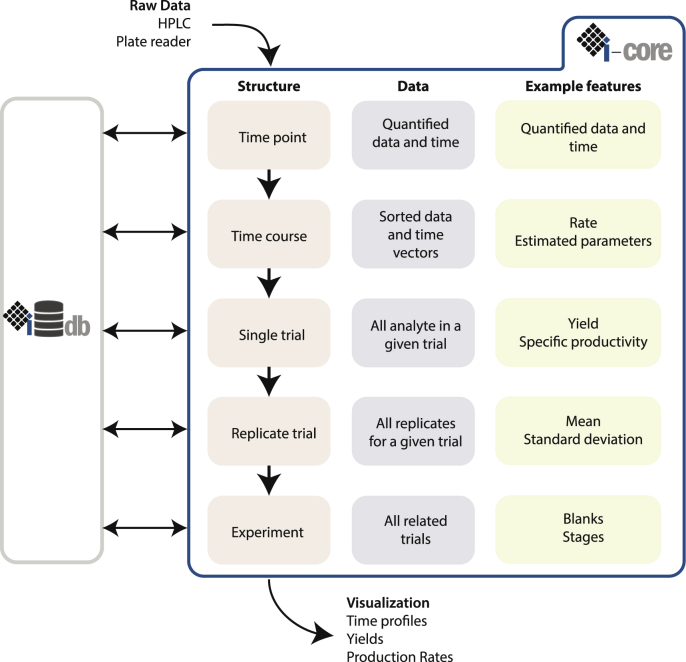
Overview of the data flow, from raw data to visualization, in the Impact framework.

The data schema of the framework (Fig. 2) is based on the logical structure of experimental design. Typically, an experiment is proposed to determine the validity of a hypothesis and this experiment will consist of a number of independent trials. Each of these trials will have a set of analytes of interest (e.g. substrate, product, and reporter) and may be performed in replicates.

• **Trial identifier** Every datum has an associated metadata component. Detailed entry of this metadata is important to ensure that the data are parsed correctly, and can then be stored and queried efficiently. New formats for trial identifiers can be created to allow more flexibility for parsing from different equipment. Currently, the trial identifier is a flat string which can be used to identify quantification events (e.g. HPLC injections) directly on analytical equipment.
• **Time point, time course, and analyte data** The most basic form of analyte data is a time point, and each analyte can have one or many time points, depending on the setup of the experiment. If several time points are present, the time points are built into a time course, which allows us to extract temporal features of the data. In addition, each analyte type (e.g. substrate, product, and reporter) inherits from this base time course class in order to implement different models to fit this data and extract unique parameters.
• **Single trial** A single trial is considered as an independent fermentation volume (e.g. a flask, or well in a plate), and thus consists of all of the analytes of interest from one trial.
• **Replicate trial** A replicate trial contains one or more single trials which are considered as replicates and can be used to extract features from these replicates such as mean and standard deviation, as well as check for consistency between replicates.
• **Experiment** Finally, an experiment contains one or more single trials in order to group relevant experiments together. A replicate trial can belong to multiple experiments, in case one trial can be used to answer multiple hypotheses.

#### 2.2.3 Object-relational mapping

A number of Python-based object-relational mapping packages exist, but the two most popular packages are Django (https://www.djangoproject.com/) and SQLAlchemy (https://www.sqlalchemy.org/). Both packages provide similar functionality, although they differ in their implementation. The simplicity of Django can be powerful, but the use of this ORM outside of a backend web application (the typical use case for Django) can be cumbersome. Instead, the Impact framework relies on SQLAlchemy because of its Pythonic syntax and portability in a range of Python environments.

### 2.3 Features

From the raw data stored in the hierarchical data schema, unique features can be extracted. As data moves up the data hierarchy, more complex features can be extracted. Analyte features are those which can be calculated with a single data vector, such as model-fitted parameters or numerically determined rates (gradients). Trial features are those which require several analytes, such as specific productivity or product yield. Replicate features are those which require multiple replicates, such as mean and standard deviation. Experiment features are typically relevant for all trials performed at a given time, such as relevant blanks or specific fermentation stages. The list of features will continue to be expanded, and new features can be added by creating, registering and committing them to the package (see documentation).

### 2.4 Availability, continuous integration, and contributing

The Impact framework is written and tested for Python ≥ 3.5 and can be installed on any system with a current Python 3 distribution.

The framework is open-source under the GPL v3 license and available on github (https://github.com/lmse/impact). Instructions for installing the framework along with all its dependencies are available on the github page for the framework. The documentation is available on readthedocs (http://impact.readthedocs.io).

The github repository has hooks connected to Travis CI (https://travis-ci.org/nvenayak/impact) to automatically run tests when new commits are merged with the repository, all new contributions should include relevant tests. The repository is also connected to codecov, to ensure that a majority of the code base is tested (https://codecov.io/gh/nvenayak/impact).

## 3 Results and discussion

### 3.1 Design, build, test, learn

**Fig. 3:**
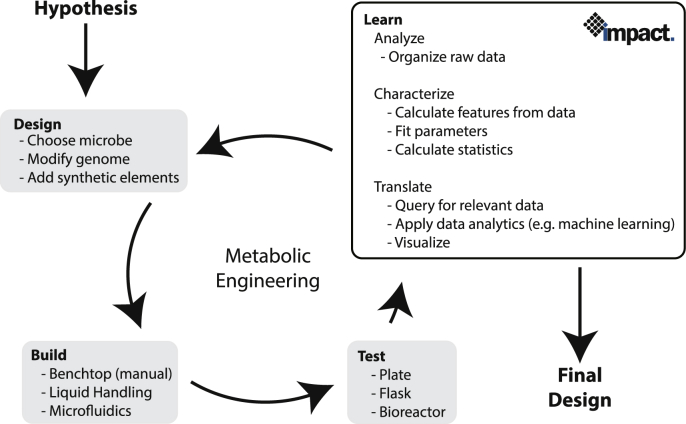
Overview of key elements in the design, built, test, learn cycle of metabolic engineering.

Metabolic engineering typically proceeds iteratively through a design-built-test-learn cycle (Fig. 3). Impact is a framework to accelerate the learning process, by automating the analysis of raw data. Thus, the framework requires raw quantified analyte data as input, which can be directly parsed from analytical equipment or a laboratory information management system (LIMS).

In brief, quantified raw data are extracted from analytical equipment without significant curation and saved into a spreadsheet (typically.xlsx). These spreadsheets can then be parsed by the Impact framework into the data schema (Fig. 2). Using this data schema, features can be extracted and finally plotted as needed. This process is divided into four stages:

1. **Analyze**: The process of parsing raw data into the data schema
2. **Characterize**: The process of extracting features or parameters from the data either directly or using a model.
3. **Translate**: The process of generating visualizations or extracting insight from raw data and calculated features.
4. **Store & share**: The process of saving data for future query by the initial user or others.

### 3.2 Analyzing raw data

#### 3.2.1 Trial identifier (metadata)

The Impact framework divides the trial identifier into three distinct components (1): the strain (2), the media, and (3) the environment. The strain describes the organism used and any genetic engineering. To do so, strains are described using a parent genus and species (the wild-type), associated knockouts, and plasmids. Each strain will of course behave differently depending on the medium used, and thus we describe the formulation of each media and associate that to a given data set. Finally, we describe the environment in which this strain was grown, including temperature, shaking speed and the labware used (microplate, flask, bioreactor, etc.)

Most effectively, this identifier should be used to label samples on primary analytical equipment (e.g. HPLC injections). Then, this data can be directly parsed by the Impact framework and raw data remaining in analytical software can be referenced. In this case, the identifier should be provided in a flat format, for example: strain:MG1655|media:M9|strain__plasmid:pTrc99a. The entire trial can be described here, or identifiers can be defined externally and referenced by shorthand name.

#### 3.2.2 Parsing raw data into the Impact framework schema

Parsing is an essential part of data interpretation, organizing raw data into logical elements which can be stored in relational tables and queried. To do so, The Impact framework makes a clear distinction between two elements: the identifier, which is responsible for metadata, and the data itself, describing the sample time and concentration. The Impact framework uses metadata to sort raw data into a hierarchical structure based on a typical experimental setup and data analysis workflow (Fig. 2).

The data structure is built around trials, which can be considered as an independent bioreactor, flask or well in a plate. Each of these trials can be composed of multiple analytes, which in turn are built from raw time and data vectors. Replicate single trials are combined to form a replicate trial object, which can then extract statistical information such as averages and standard deviations. This organization process is handled solely by the parser, and the user typically will not need to modify this process except to handle new raw data formats.

#### 3.2.3 Data formats

Data describing microbial phenotypes are varied, depending on the particular attributes of interest for a given project. Here we provide parsers for two common pieces of equipment: HPLC and plate reader. The HPLC is a workhorse in metabolic engineering, providing the external concentration of diverse metabolites which can be readily be parsed by the Impact framework (Table S1). Apart from its widespread use in enzyme assays, the plate reader is commonly used to measure growth profiles via optical density, or specific aspects of metabolism using fluorescent probes, and we provide parsers for data from SoftMax Pro (Table S2) and more typical readers (Table S3).

Depending on the complexity of the data set, parsers are generally simple to write and register to the framework. The raw data are parsed into a basal data type (e.g. a time point), and then standard parsing functions organize the data into the appropriate data structures. The process of creating a new parser is described in detail in the documentation.

### 3.3 Characterizing physiological features

The Impact framework provides a set of core features, which are derived from the raw quantified data. These features are extracted at different stages, corresponding to the data required. A set of features is provided in the current version, and new features can be created as needed and contributed back to the package.

#### 3.3.1 Analyte features

Analyte features are those which can be calculated with only one analyte.

Although state metrics, such as yield and titer, are often used to describe microbial phenotypes, dynamic metrics such as rates and specific productivities can provide further insight. These rates are determined numerically, for each analyte, and used for subsequent calculations. Alternatively, rates can be extracted via parameter fitting.

Typically microbial growth kinetics are described as exponential: $\frac{dX}{dt}=\mu X\Leftrightarrow X=X_{0}e^{\mu t}$. However, since microbial growth is oftentimes characterized through lag, log, and death phases, high order models may be relevant. Any model can be added for analysis, and some common models such as the 5-parameter Richards curve or a generalized logistic function (Zwietering et al., 1990) are included.

#### 3.3.2 Trial features

Trial features are calculated from a single fermentation volume but require several analytes. These features are widespread, and examples include product yield (which requires a product and substrate), OD normalization (normalization of data to cell density) or specific productivity (which require biomass and an additional analyte).

The specific productivity is the unit most often used in constraint-based models. It is defined as the rate of product export per unit of biomass, usually with the units $\frac{mmol}{gdw\;h}$ ($h^{-1}$ in the case of growth rate) and can be directly used to constrain metabolic models.

#### 3.3.3 Replicate features

Experimental replicates are typically used to ensure consistency in data and conclusions. Thus, if multiple replicates are available, they are combined to calculate statistical features. If no replicates are available, the *mean* is calculated from a single replicate. With small data sets, outlier experiments can be easily identified and excluded. However, with large quantities of data, this becomes challenging and can drastically affect data interpretation. Thus, outliers can be detected as replicates which deviate significantly from othersand be excluded from analysis. Parameters can be chosen to control the aggressiveness of this process.

#### 3.3.4 Experiment features

Experiment features are those which are relevant to many independent trials, such as the different stages in a production batch, or blanks. Oftentimes, there can be a benefit of dividing a fermentation into distinct stages, while optimizing different process parameters at each (Venayak et al., 2015; Soma et al., 2014; Cress et al., 2015; Brockman and Prather, 2015). Analyzing each of these stages independently is often necessary to extract more relevant metrics. For example, product yields can be more insightful when only considering the production stage.

Blank or background subtraction is a common process to eliminate background signal for more accurate quantification and can automatically be performed. Typically, specific trials which do not contain cells can be used as blanks, and these trials can automatically be assigned to non-blank trials for subtraction.

### 3.4 Translating and interpreting data

#### 3.4.1 Visualization

**Fig. 4:**
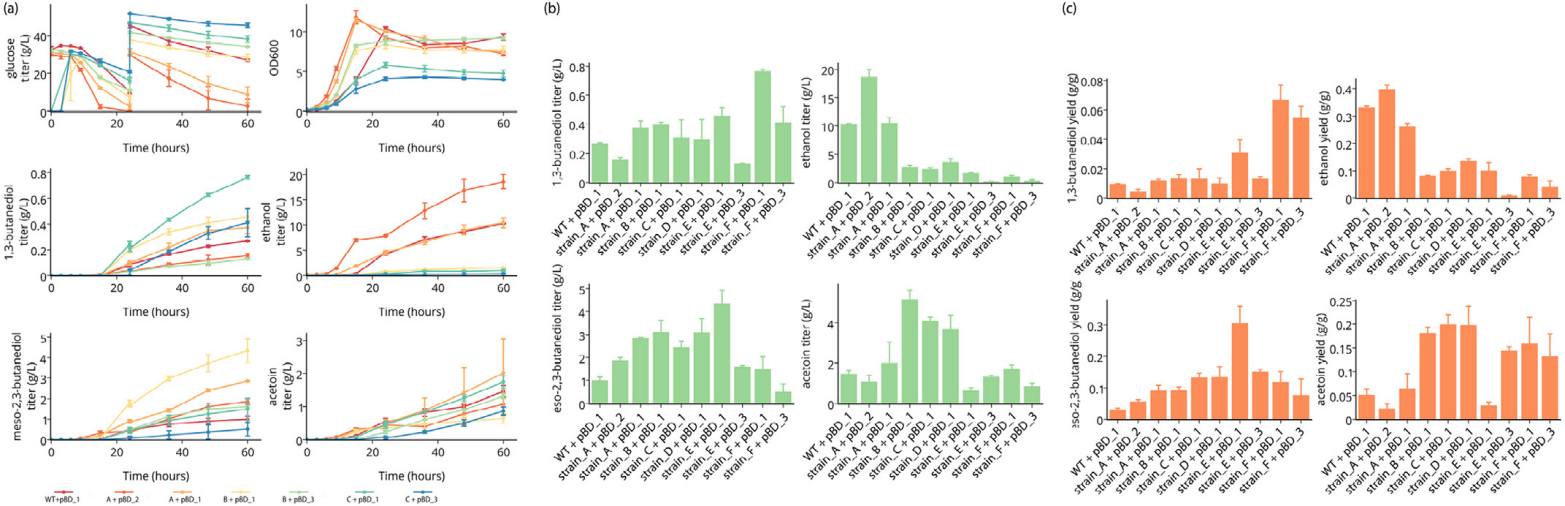
Sample data visualization for a metabolic engineering problem generated with the Impact framework - (a) time course data (b) titer (c) yield. Data adapted from Nemr et al., 2018 where the aim was to develop a platform strain to produce 1,3-butanediol. Such a comparison of yields, titers, and productivities of different microbial strains could help scientists decide on appropriate intervention strategies to improve the metric of their choice. For example, the data analysis by the Impact framework seems to suggest that “strain_E” with the plasmid “pBD_3” has the highest end point titer of 1,3-butanediol (shown in panel ‘b’) while having a significantly lower yield than other strains as seen in panel ‘c’. The Impact framework can perform this analysis and visualize the data within a few seconds while analysis of the data, calculation of statistical information and plotting the processed data manually might take several hours.

By default, visualization is accomplished by defining subplots per analyte and plotting the average data for each strain. Using this format, figures ready for interpretation can easily be generated. The Impact framework comes built with some basic plotting routines that assist the user to generate time-course plots of analytes (e.g. substrate, product, and reporter) or their features (e.g. biomass specific productivity). In lieu of the provided options, figures can be generated directly using any number of Python plotting packages such as Matplotlib (Hunter, 2007) (Fig. 4).

**Fig. 5:**
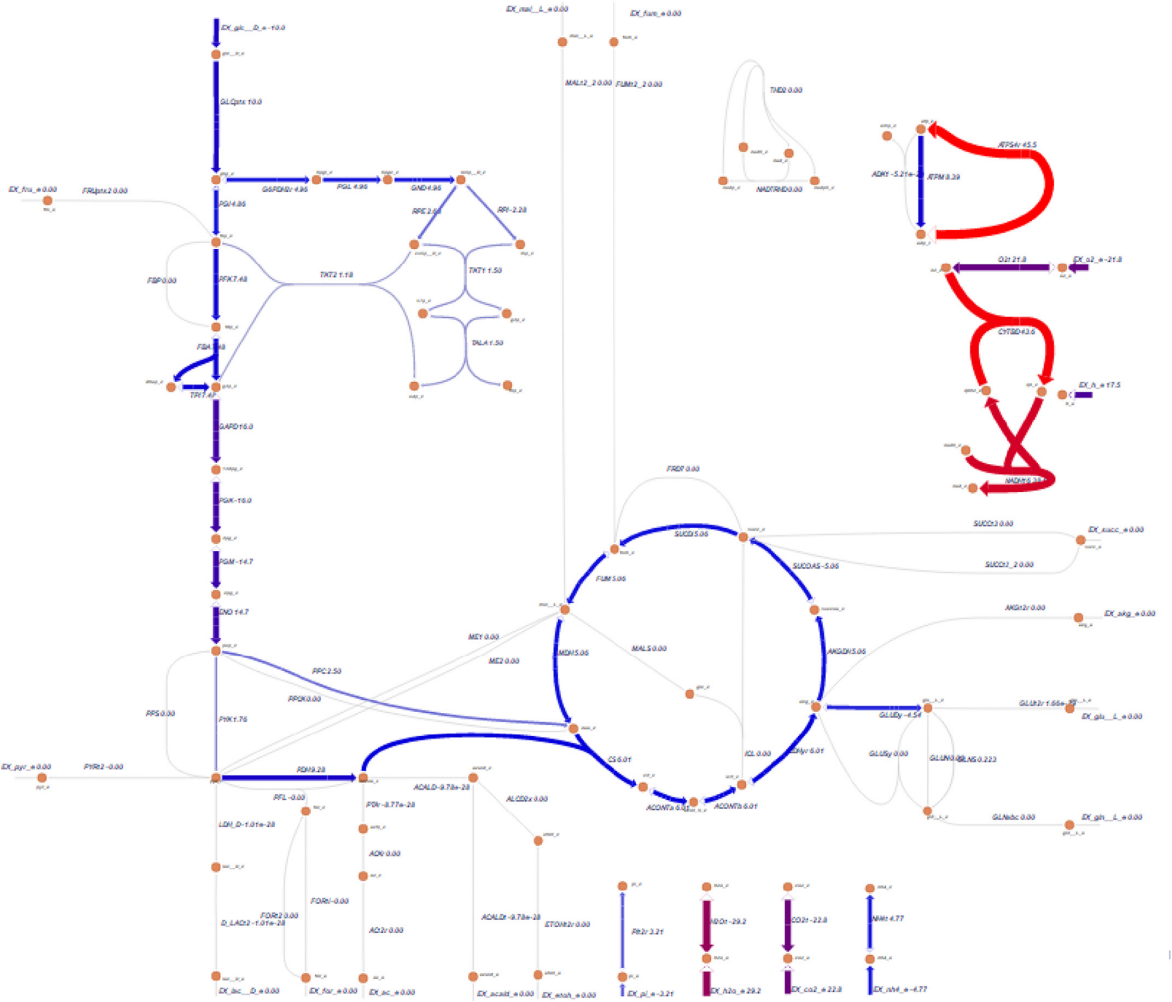
Sample data visualization generated with Escher with data from the Impact framework.

In addition, this data can be used to constrain a metabolic model and plot fluxes using other packages such as Escher (King et al., 2015a), a Python based flux visualization package (Fig. 5).

#### 3.4.2 Mass balance

**Fig. 6:**
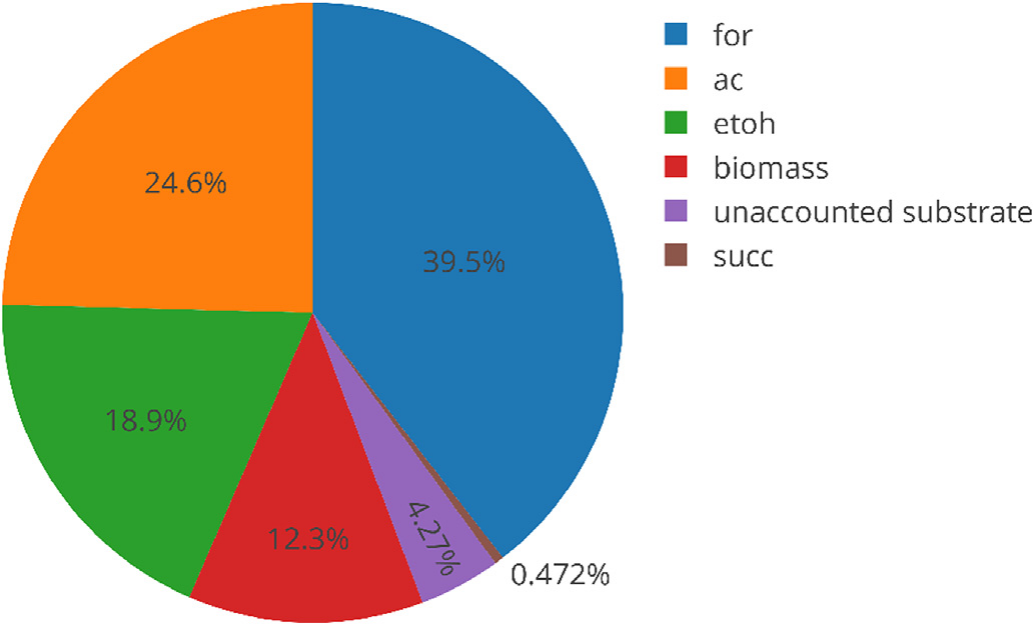
Carbon balance using an anaerobic *E. coli* simulation with iJO1366. for: formate, ac: acetate, etoh: ethanol, succ: succinate.

A carbon balance or mass balance can be important to ensure that all significant analytes are accounted for, including gaseous and liquid phases. Oftentimes gaseous products are not analyzed, in which case CO_2_ can be estimated using an integrated metabolic model constrained using known production and consumption rates (Fig. 6).

#### 3.4.3 Missing data

Missing data pose a significant challenge for many data analysis tasks. Missing data can be relatively common due to experimental or equipment error. To overcome this limitation, we take advantage of the Pandas package (https://pandas.pydata.org/), where data input with ’nan’ values will not be included in statistical calculations and data sets with different time indices are handled seamlessly (Table 1).

**Table 1:** Example of missing data handling included with the Impact framework through the Pandas package.

time	0	2	4	6
replicate #1	✓ 0.5	✓ 0.8	✓ 0.9	✓ 1.2
replicate #2	✓ 0.6	✗ nan	✓ 1.0	✗ nan
replicate #3	✓ 0.4	✓ 0.7	✓ 1.2	✗ nan
average	0.5	0.75	1.03	1.2
standard deviation	0.1	0.07	0.15	n/a

#### 3.4.4 Metabolic model integration

Using the aforementioned calculated specific productivities, basic integration with metabolic models is relatively straightforward. A purely stoichiometric metabolic model can be solved by constraining only the substrate uptake rate and using an objective function to estimate the flux distribution. With experimental data, we can follow a similar approach but add additional constraints for all measured analytes and estimate internal fluxes for additional insight into metabolism.

### 3.5 Data storage and retrieval

#### 3.5.1 The object relational mapping

Although organization of raw data into a logical structure eases data interpretation and visualization, sharing and collaboration are still limited. Since the process of biological engineering is highly collaborative and relies on data from a large number of scientists and engineers, this is of paramount importance. The Impact framework aims to store all this data in a standardized database, so it can be shared and queried.

Relational databases have become the workhorse for most data storage tasks since they are highly structured and can be efficiently queried. To avoid the complexity and domain knowledge associated with such data structures, the Impact framework is built on top of an object relational mapping (ORM) framework, SQLAlchemy. This allows developers to focus on adding new functionality using a familiar programming language (Python) and programming paradigm (object oriented programming), while still benefiting from the structured query language of a relational database.

#### 3.5.2 Narratives and the Jupyter notebook

The rate of data collection and the complexity of data analyses continue to increase. Narratives allow scientists to disseminate complex information by providing context and guiding their audience through experiment setup, analysis, and the relevant conclusions. Jupyter notebooks provide the perfect platform for this type of narrative in a programming context and as such is receiving significant interest (Shen, 2014). In addition to writing and sharing Jupyter notebooks locally, significant progress has been made to deploy this platform on the cloud, allowing scientists and engineers to begin writing narratives without any local installation (KBase - https://kbase.us, JupyterHub - https://github.com/jupyterhub/jupyterhub).

## 4 Conclusion

The increasing application of microbial solutions to diverse challenges has drastically accelerated the rate of data generation. Engineering these microorganisms is a highly iterative process through the design-build-test-learn cycle, and evolving tools are continuing to reduce the cycle time. Here, we present the Impact framework, a Python package aimed to unify stakeholders in the bioengineering process and provide a set of tools to characterize microbial physiology using programmatic workflows. The architecture of the Impact framework is aimed to make community contribution simple and make complex data workflows more transparent and shareable. We expect that the application of such tools following software development best practices (Yurkovich et al., 2017) to continue to develop and accelerate the process of microbial engineering.
