# Supplementary material for: Impact framework: A python package for writing data analysis workflows to interpret microbial physiology
Source: Metab Eng Commun. 2019 Apr 4;9:e00089. doi: 10.1016/j.mec.2019.e00089 (PMC6462781; doi:10.1016/j.mec.2019.e00089)
Supplement: Multimedia component 1 [file mmc1.zip › Supporting_Material/QuickStart.pdf]

In [1]:

```
import impact as impt
import os
```

```
C:\Users\kraj5\Anaconda3\lib\site-packages\IPython\html.py:14: ShimWarning:
The `IPython.html` package has been deprecated since IPython 4.0. You should
import from `notebook` instead. `IPython.html.widgets` has moved to `ipywidg
ets`.
  "`IPython.html.widgets` has moved to `ipywidgets`.", ShimWarning)
```

The impact framework is designed to help scientists parse, interpret, explore and visualize data to understand and engineer micorbial physiology. The core framework is open-source and written entirely in python. The repository along with documentation is available on https://github.com/nvenayak/impact (https://github.com/nvenayak/impact).

Data is parsed into an object-oriented data structure, built on top of a relational mapping to most sql databases. This allows for efficient saving and querying to ease data exploration.

Here we provide the basics to get started analyzing data with the core framework. Before getting started, it is worthwhile to understand the basic data schema:

Model	Function
TrialIdentifier	Describes a trial (time, analyte, strain, media, etc.)
AnalyteData	(time, data) points and vectors for quantified data (g/L product, OD, etc.)
SingleTrial	All analytes for a given unit (e.g. a tube, well on plate, bioreactor, etc.)
ReplicateTrial	Contains a set of `SingleTrial` s with replicates grouped to calculate statistics
Experiment	All of the trials performed on a given date

On import, data will automatically be parsed into this format. In addition, data will most commonly be queried by metadata in the `TrialIdentifier` which is composed of three main identifiers:

Model	Function
Strain	Describes the organism being characterized (e.g. strain, knockouts, plasmids, etc.)
Media	Described the medium used to characterize the organism (e.g. M9 + 0.02% glc_D)
Environment	The conditions and labware used (e.g. 96-well plate, 250RPM, 37C)

Importing data

Data is imported using the `parse_raw_data` method of the `Parser` class from the `impact.parsers` module. This function returns an `Experiment` , which is the result of organizing all of your data.

To parse data, the data is usually provided in an xlsx file in one of the desired formats. If your data doesn't conform to one of the built-in formats, you can use the provided parsers as a cookbook to build your own. Generally, minor edits are required to conform to new data.

Here we use the sample test data, which is a typical format for data from HPLC. Each row is a specific trial and time points, and the columns represent the different analytes, and their types. You can see this data in `sample_data/Fermentation_1_impact.xlsx`

In [2]:

```
from impact.parsers import Parser
from pprint import pprint
expt = Parser.parse_raw_data('default_titers',
                             file_name = os.path.join('sample_data','Fermentation_1_impac
                             id_type='traverse')
expt.calculate()
```

```
Importing data from sample_data\Fermentation_1_impact.xlsx...0.0s
Parsed 600 timeCourseObjects in 0.542s...Number of lines skipped:  0
Parsing time point list...Parsed 600 time points in 0.1s
Parsing analyte list...Parsed 6 single trials in 89.8ms
Parsing single trial list...Parsed 2 replicates in 0.0s
Analyzing data...No blanks were indicated. Blank subtraction will not be don
e.Ran analysis in 0.1s


c:\users\kraj5\onedrive\research\phd_uoft\paper_submissions\impact_mec\impac
t\impact\core\features\ProductYield.py:22: RuntimeWarning: invalid value enc
ountered in true_divide
  self.substrate_consumed
```

The data is now imported and organized, we can quickly get an overview of what we've imported.

In [3]:

```
print(expt)
```

```
strain                          media                   environment    analyte
s
----------------------------  ----------------------  -------------  -------
----------------------------------------------------------------------------
-
BD2 Δ(adhE,ldhA,pflB) + pBD1  mod M9 + 1.0 a.u. IPTG  None           ['acal
d', 'acet', 'od', 'suc', 'm23bdo', 'glc__D', '13bdo', 'rs23bdo', 'etoh', 'a
c']
BD4 Δ(adhE,ldhA,pflB) + pBD3  mod M9 + 1.0 a.u. IPTG  None           ['acal
d', 'acet', 'od', 'suc', 'm23bdo', 'glc__D', '13bdo', 'rs23bdo', 'etoh', 'a
c']
```

Before we dive into data analysis, it is worth having a basic understanding of the schema to know where to look for data.

Firstly, all data is funneled into a `ReplicateTrial` , even if you only have one replicate. As such, it is convenient to always look for data in this object. This object contains both an `avg` and `std` attribute where you can find the respective statistics. `avg` and `std` attributes are instances of `SingleTrial` , so we can access the statistical data similarly to the raw data itself.

Querying and filtering for data

After import, data is all sorted into python objects, associated to an sql database using an object-relational mapper, SQLalchemy. Usually, we're interested in comparing a set of features and a set of conditions (strain, media, environment) and the queryable database allows us to search for the data we are interested in.

Although it is usually simple to use the ORM to access the database directly, basic querying can also be done using python list comprehensions. The major limitation is that you will only query experiments loaded in memory, e.g. experiments that were parsed into this notebook.

In [4]:

```
reps = [rep for rep in expt.replicate_trials]
for rep in reps:
    print(rep.trial_identifier)
```

```
strain: BD2 Δ(adhE,ldhA,pflB) + pBD1,   media: mod M9 + 1.0 a.u. IPTG,   env:
None
strain: BD4 Δ(adhE,ldhA,pflB) + pBD3,   media: mod M9 + 1.0 a.u. IPTG,   env:
None
```

In [5]:

```
reps = [rep for rep in expt.replicate_trials
       if rep.trial_identifier.strain.name == 'BD2']
for rep in reps:
    print(rep.trial_identifier)
```

```
strain: BD2 Δ(adhE,ldhA,pflB) + pBD1,   media: mod M9 + 1.0 a.u. IPTG,   env:
None
```

To use the database, we must query data through a `session` object. The session is open for the entire application.

In [6]:

```
session = impt.database.create_session()
engine = impt.database.bind_engine()
impt.database.Base.metadata.create_all(engine)
```

Now that we have a session, we can use the standard SQLalchemy ORM language to add elements or query - it is described in detail here [http://docs.sqlalchemy.org/en/latest/orm/tutorial.html#querying (http://docs.sqlalchemy.org/en/latest/orm/tutorial.html#querying)](http://docs.sqlalchemy.org/en/latest/orm/tutorial.html#querying)

Let us add the experiment to our database and then delete it from memory

In [7]:

```
session.add(expt)
session.commit()
del expt
```

We will attempt to retrieve elements from the database. We will look for replicate trials which use the strain BD2 as follows and print the trial_identifier for that replicate trial. We also retrieve the experiment object for further use in this document.

In [8]:

```
reps = session.query(impt.ReplicateTrial)\
                .join(impt.ReplicateTrialIdentifier)\
                .join(impt.Strain)\
                .filter(impt.Strain.name == 'BD2').all()

for rep in reps:
    print(rep.trial_identifier)

expt = session.query(impt.Experiment).all()[0]
```

```
strain: BD2 Δ(adhE,ldhA,pflB) + pBD1,   media: mod M9 + 1.0 a.u. IPTG,  env: 
None
```

Visualization

Several packages already exist for visualization in python. The most popular one in matplotlib, it has very simple syntax which should feel familiar for matlab users; however, matplotlib generates static plots. The Impact visualization module is built around plotly, which generated dynamic javascript plots, and as such it is worthwhile understanding the basic syntax of plotly charts.

In [9]:

```
import impact.plotting as implot
import numpy as np

# Charts are made up in a hierarchical structure, but can be quickly generated as follows
x = np.linspace(0,10,10)
y = np.linspace(0,10,10)**2
implot.plot([implot.go.Scatter(x=x,y=y),
             implot.go.Scatter(x=x,y=y*2)])

# For more control over these plots, they can be built form the ground up
# Traces are defined for each feature
traces = [implot.go.Scatter(x=x,y=y),
             implot.go.Scatter(x=x,y=y*2)]

layout = implot.go.Layout(height=400,width=400)

# Traces are joined to a figure
fig = implot.go.Figure(data=traces, layout=layout)

# And a figure is printed using plot
implot.plot(fig)
```

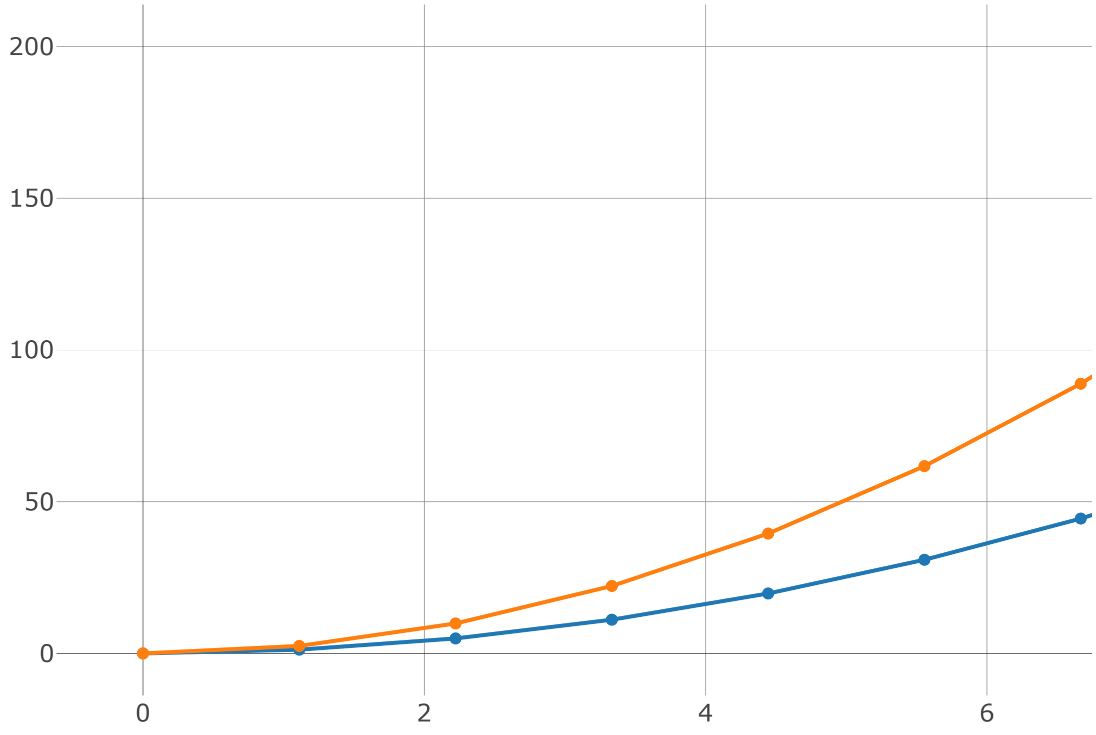

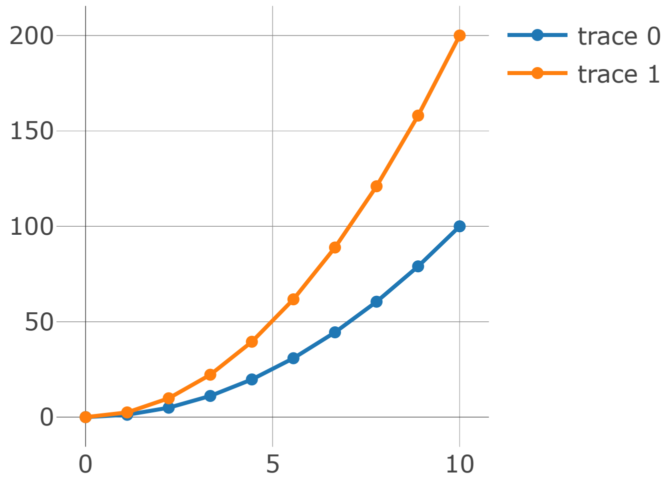

Export to plot.ly »

It should be noted that the implot package offers a direct wrapper to useful plotly functions, which could also be accessed with plotly directly. The Impact visualization module offers functions to help extract useful data and generate traces. Below, we generate plots of analyte titer vs time for each analyte. Impact comes built with plotting routines that generate seperate plots of timecourses/max. values, with experiments separated by strain name, knockouts, plasmids, media and media components.

In [10]:

```
implot.plot_timecourse_orderby_basemedia(expt)
```

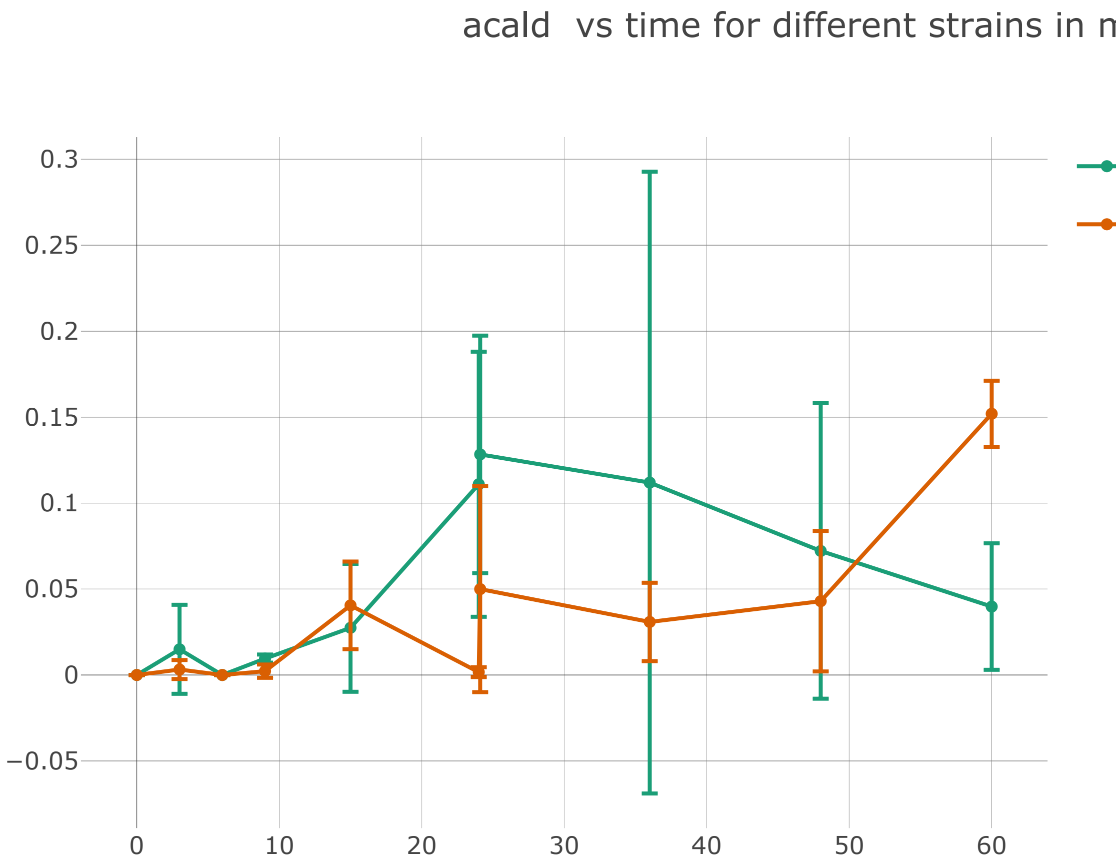

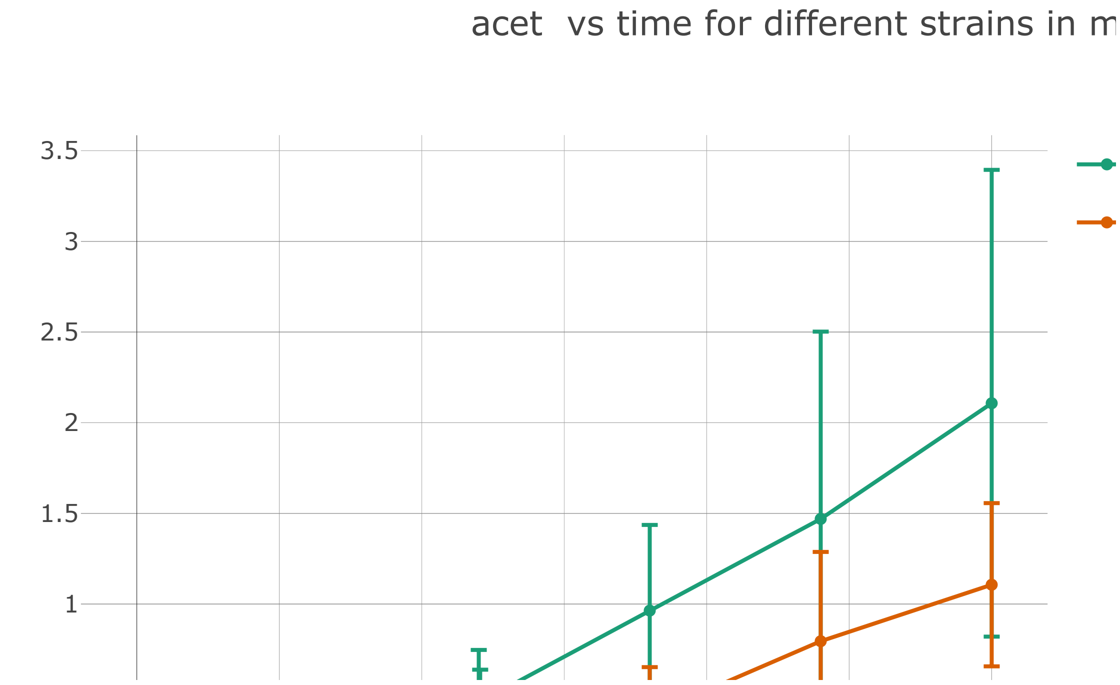

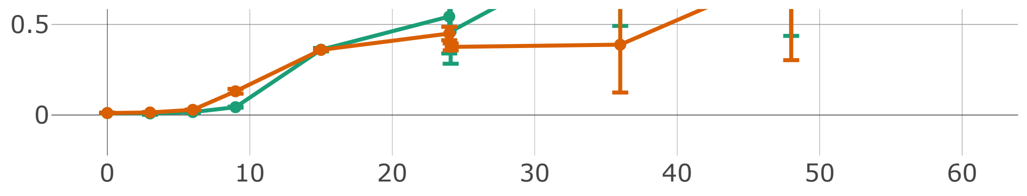

od vs time for different strains in mo

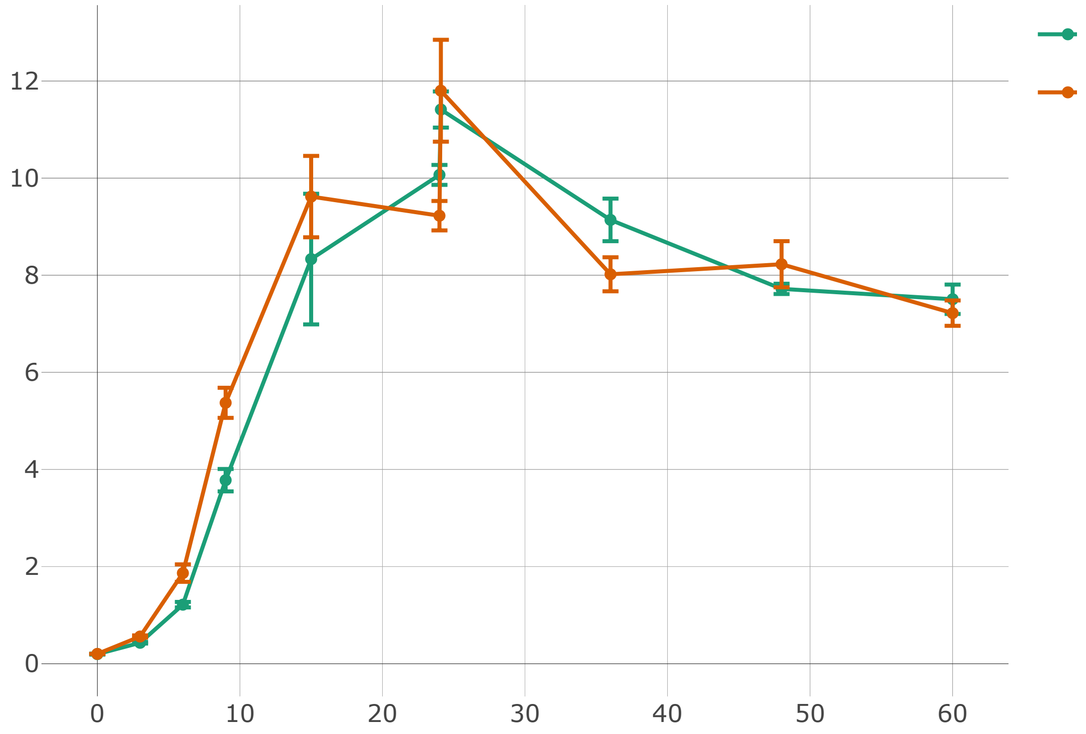

suc vs time for different strains in m

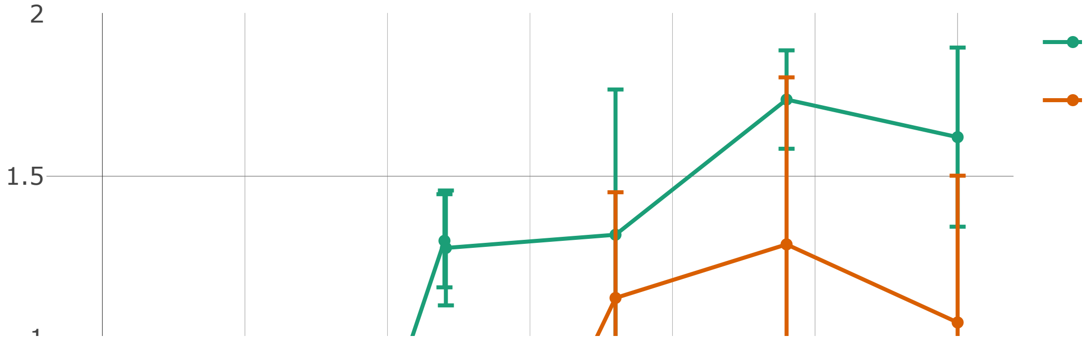

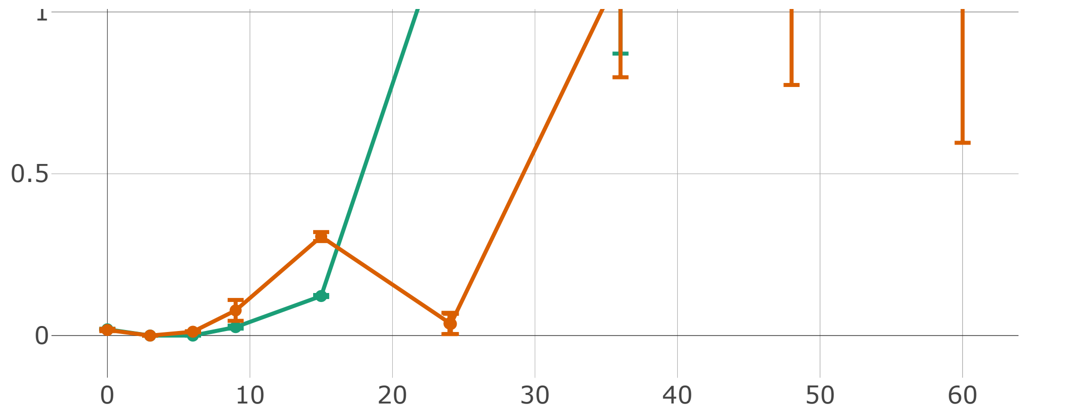

m23bdo vs time for different strains in

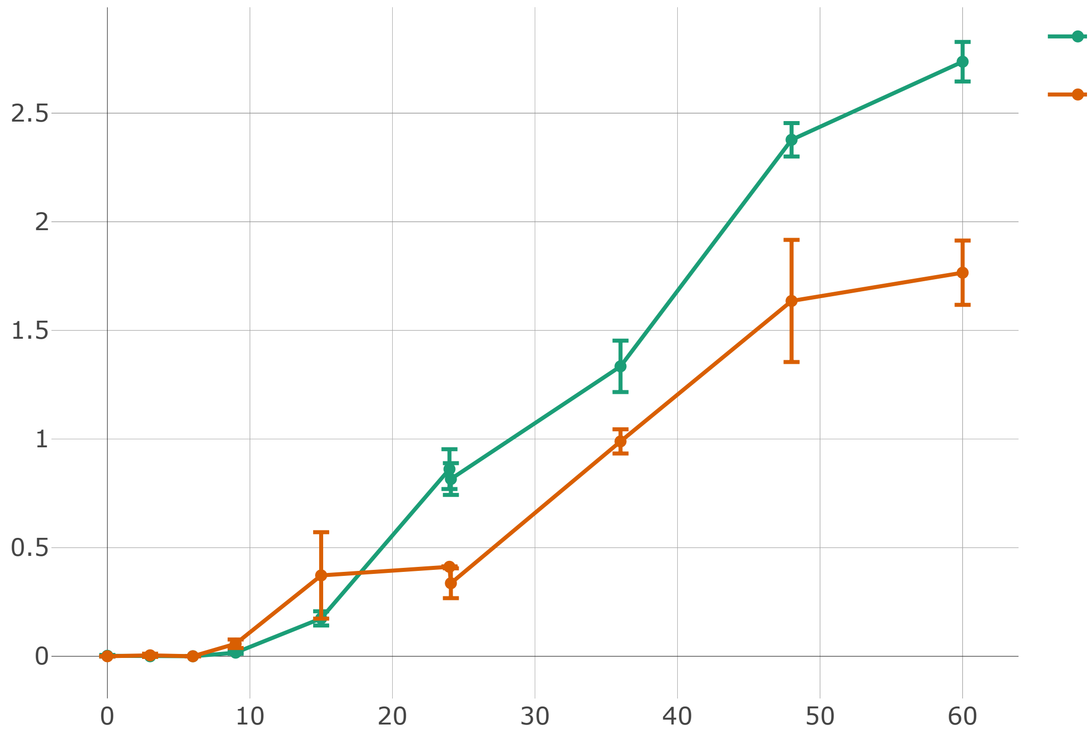

glc__D vs time for different strains in

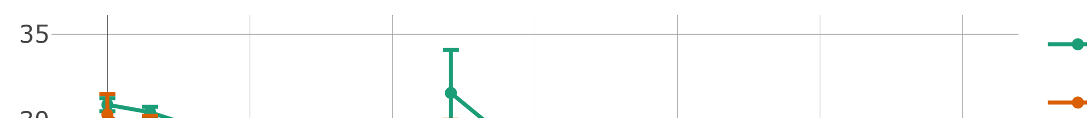

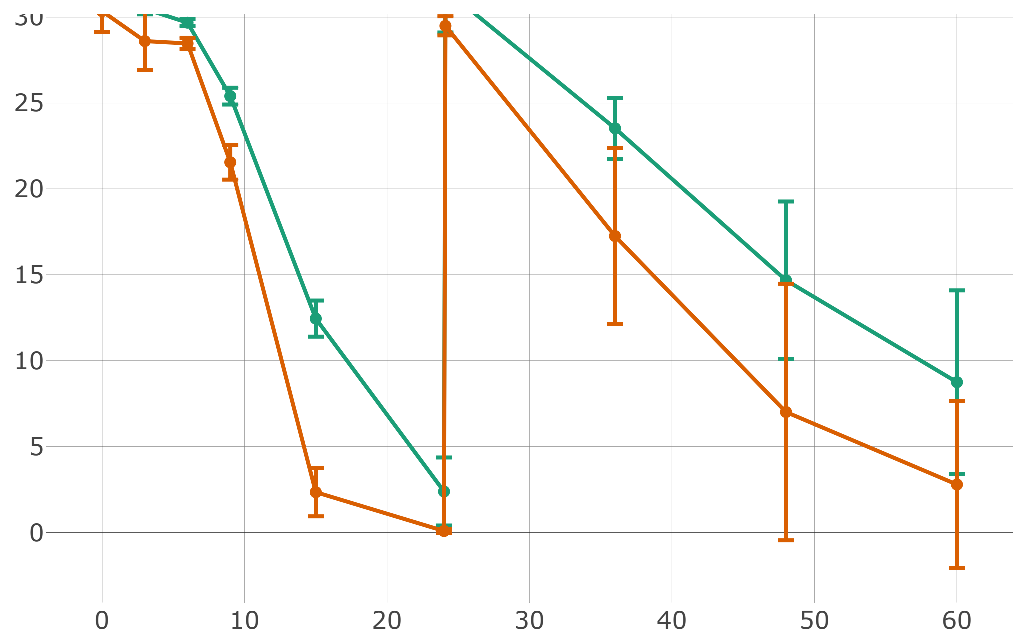

13bdo vs time for different strains in r

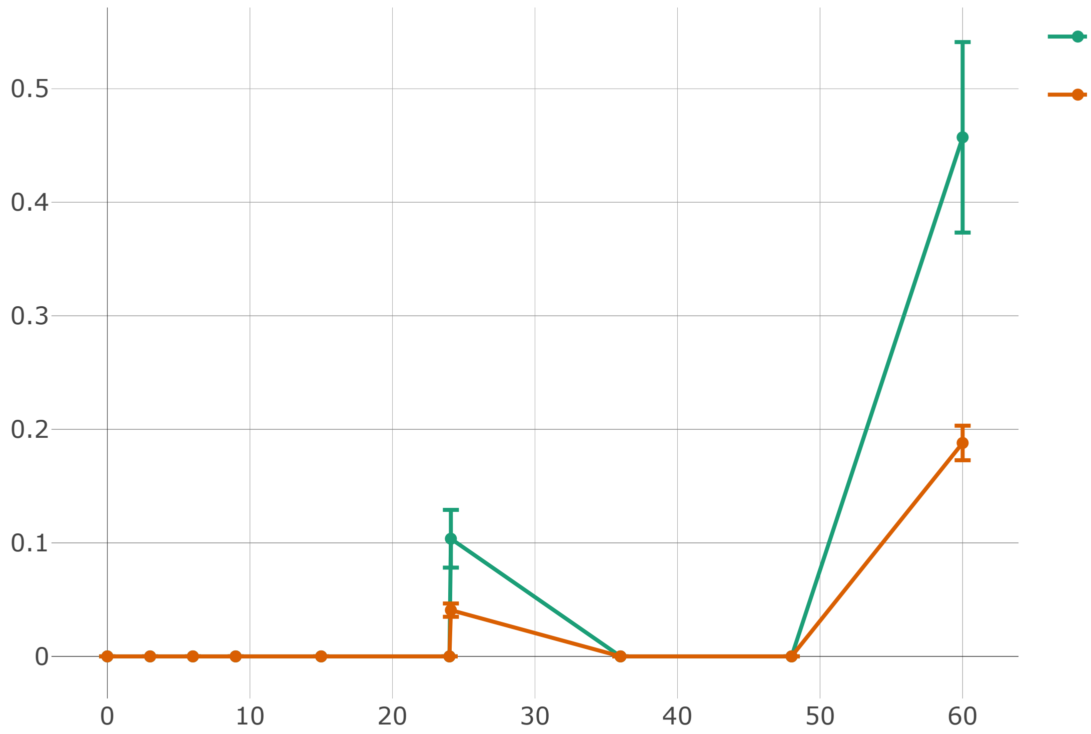

[illegible]23bdo vs time for different strains in

rs23bdo vs time for different strains in

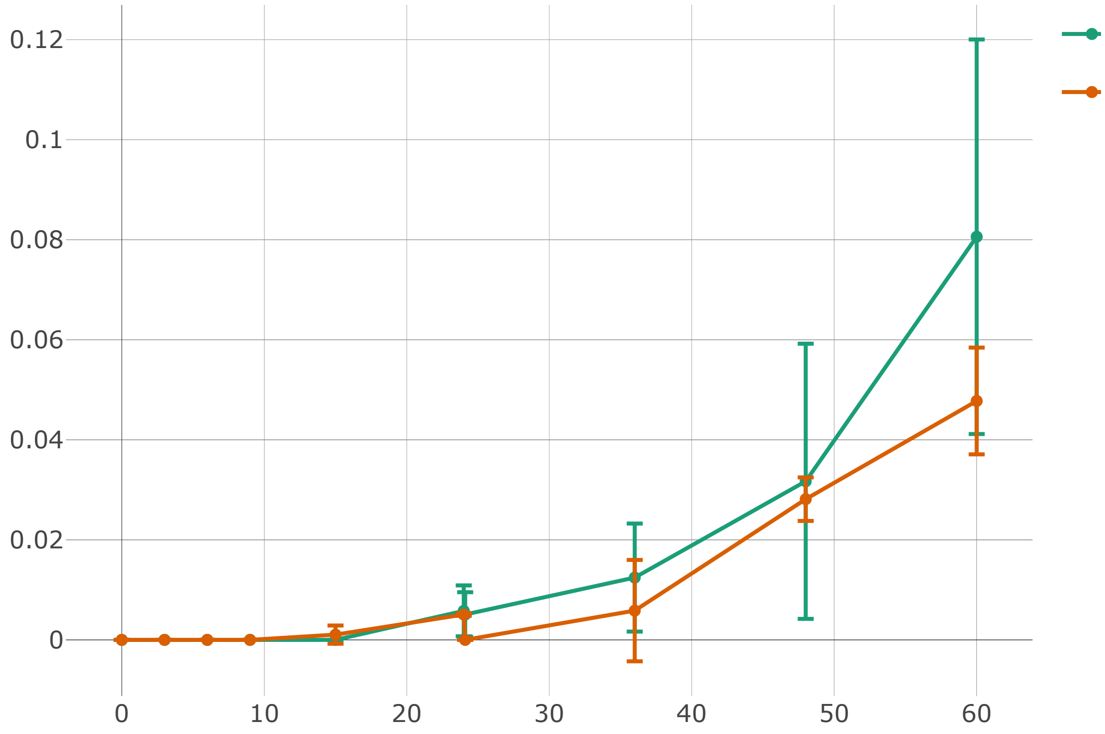

etoh vs time for different strains in m

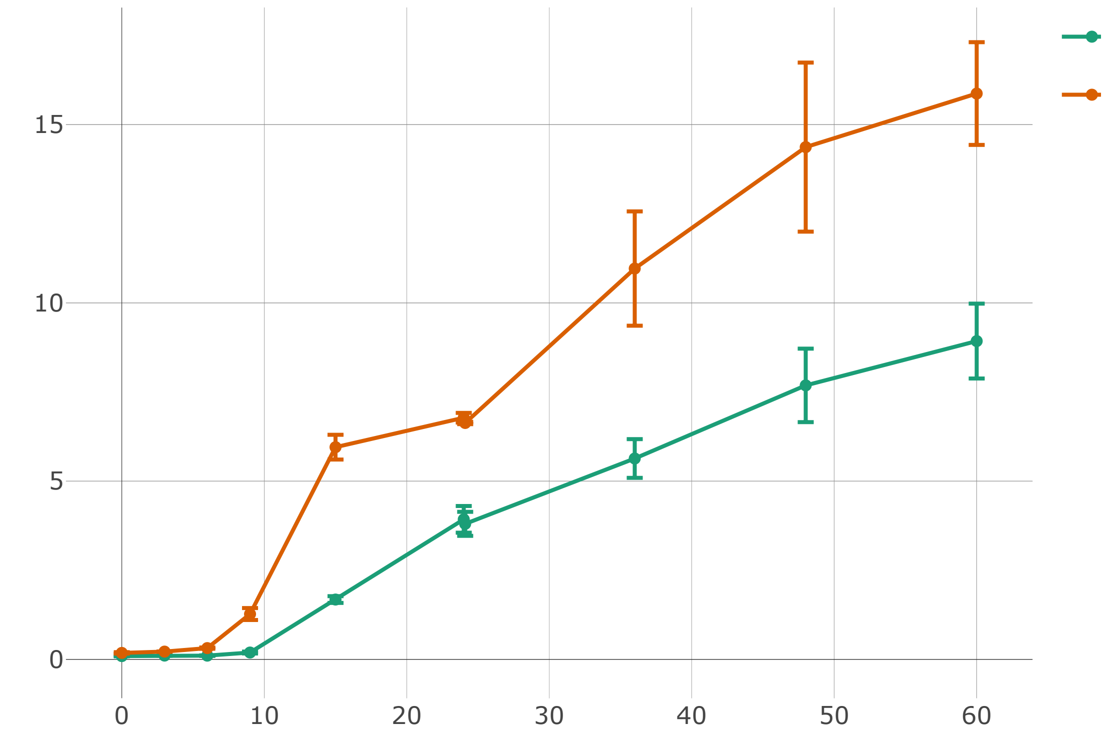

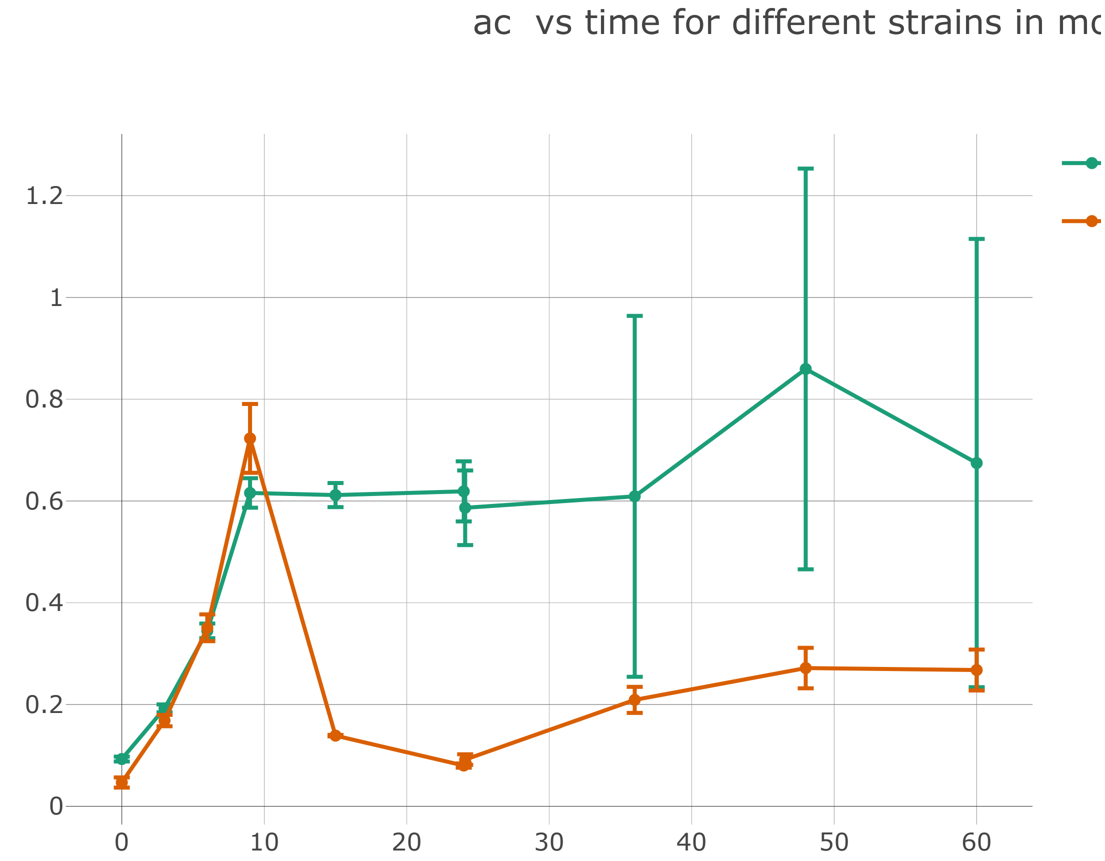

Exploring features

With a standard schema for the data, we can now begin to explore some of the features which have been generated. Features include things like:

- rate ($g \quad h^{-1}$)
- yield ($g_{product} \quad g_{substrate}^{-1}$)
- specific productivity ($g \quad gdw^{-1} \quad h^{-1}$)
- normalized data (e.g. $a.u.fluorescence \quad OD_{600}^{-1}$)

In [11]:

```
implot.plot_timecourse_orderby_basemedia(expt,feature='specific_productivity')
```

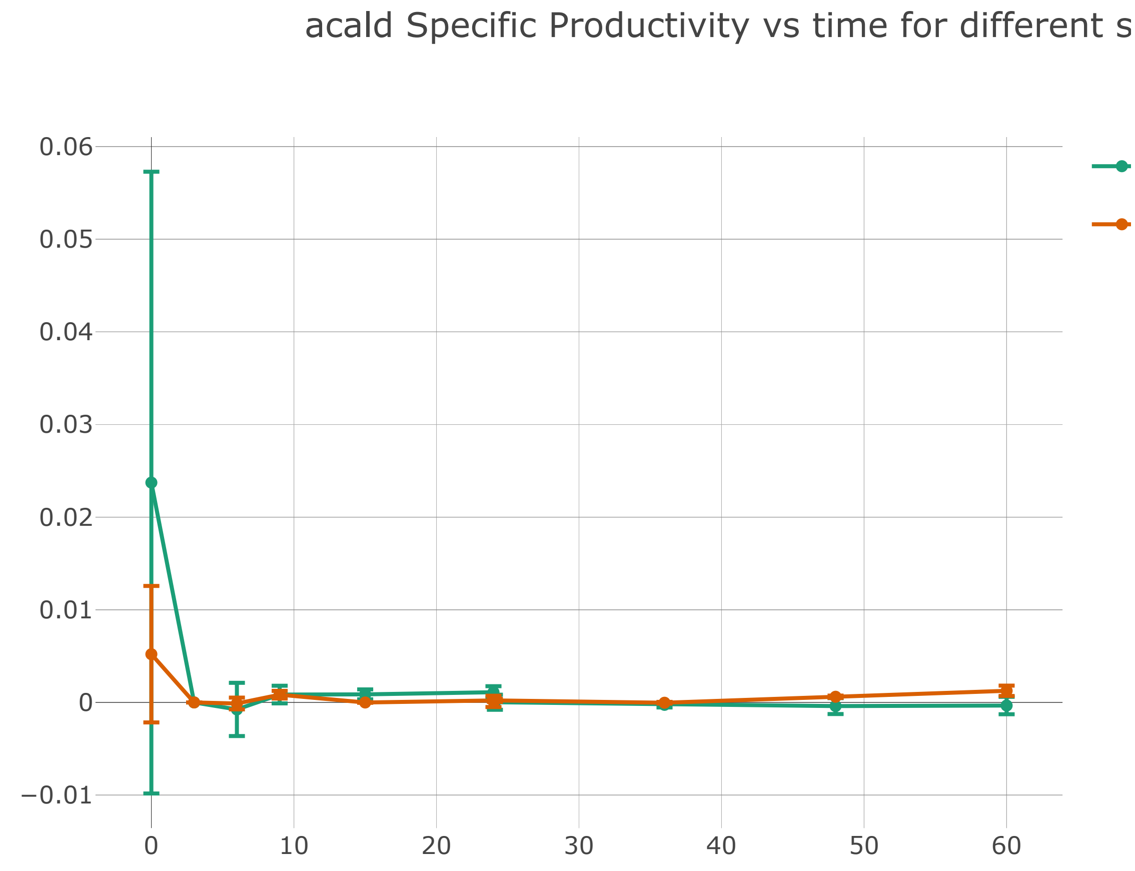

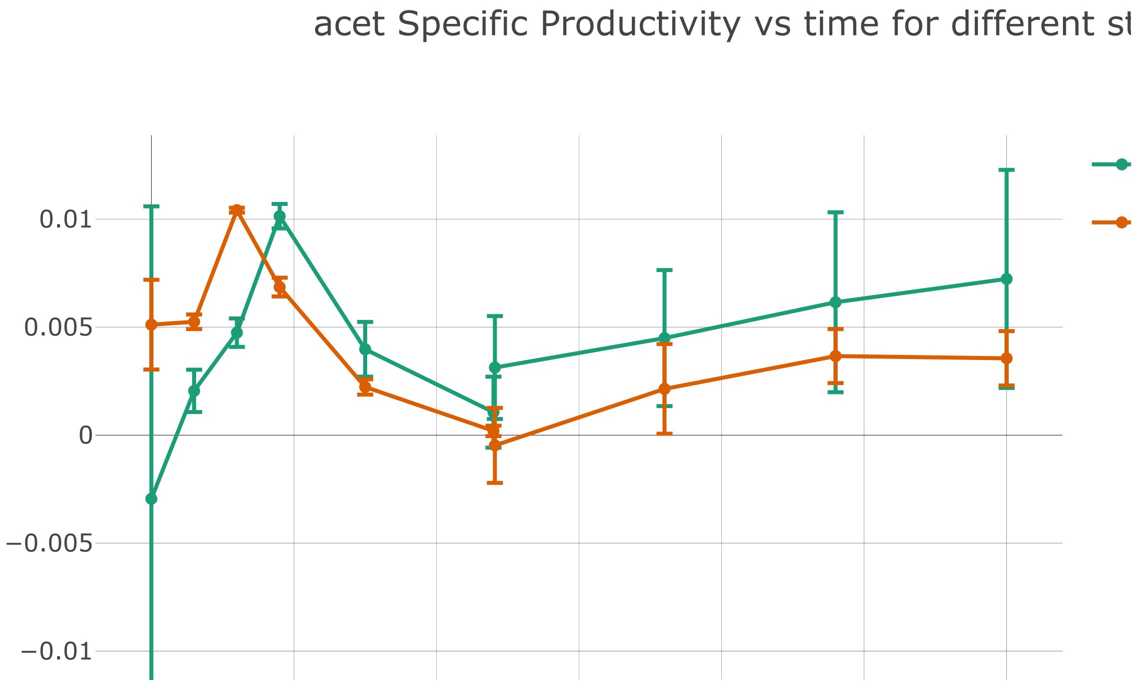

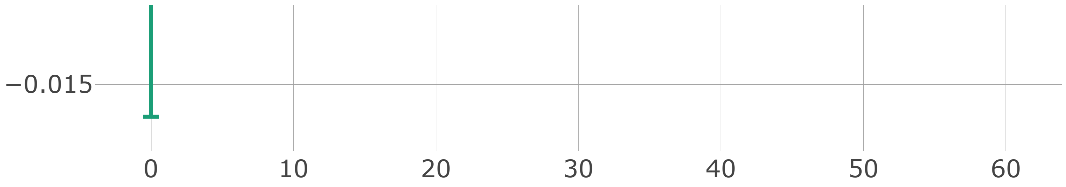

−0.015
0
10
20
30
40
50
60

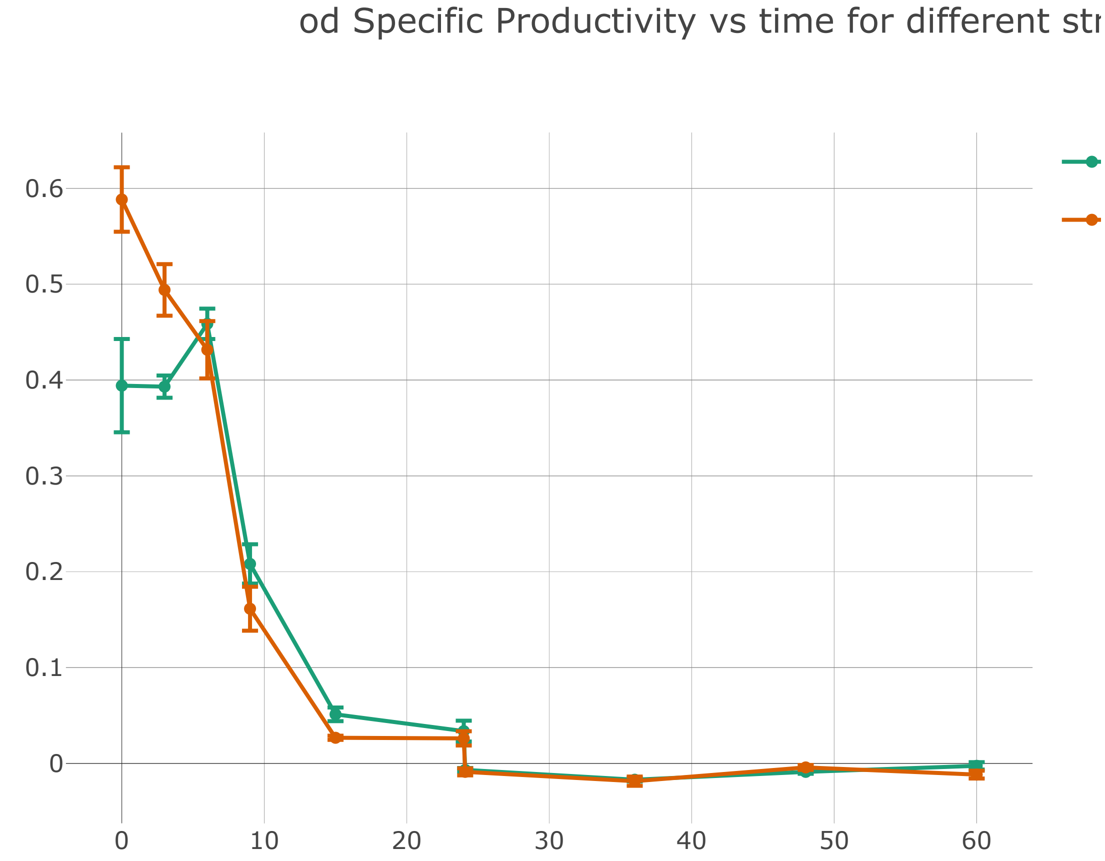

od Specific Productivity vs time for different str
0.6
0.5
0.4
0.3
0.2
0.1
0
0
10
20
30
40
50
60

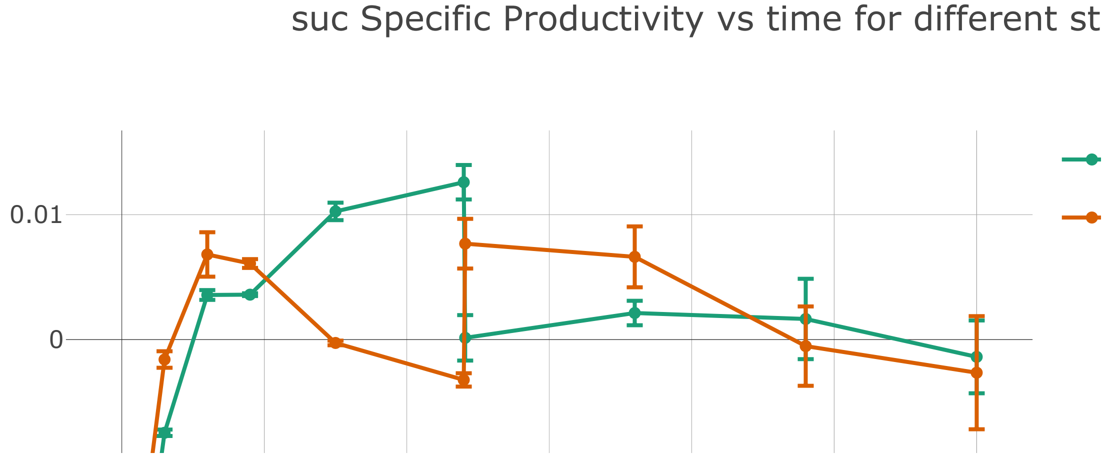

suc Specific Productivity vs time for different st
0.01
0

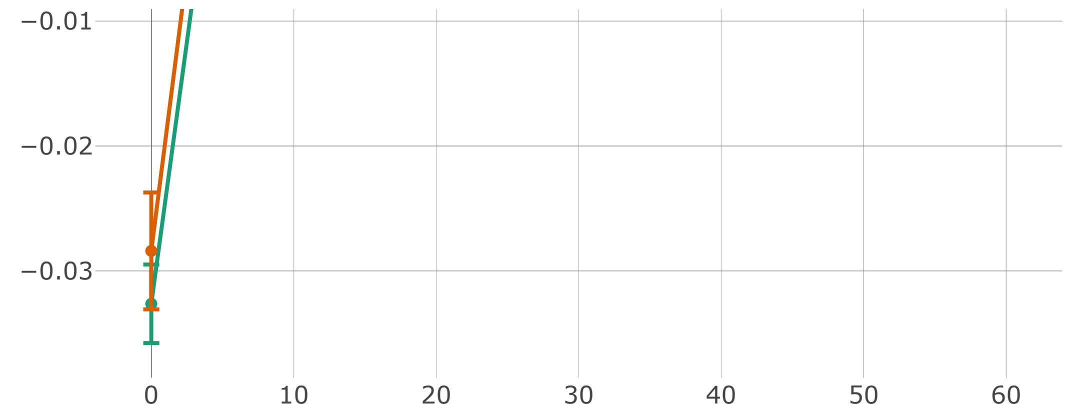

m23bdo Specific Productivity vs time for different

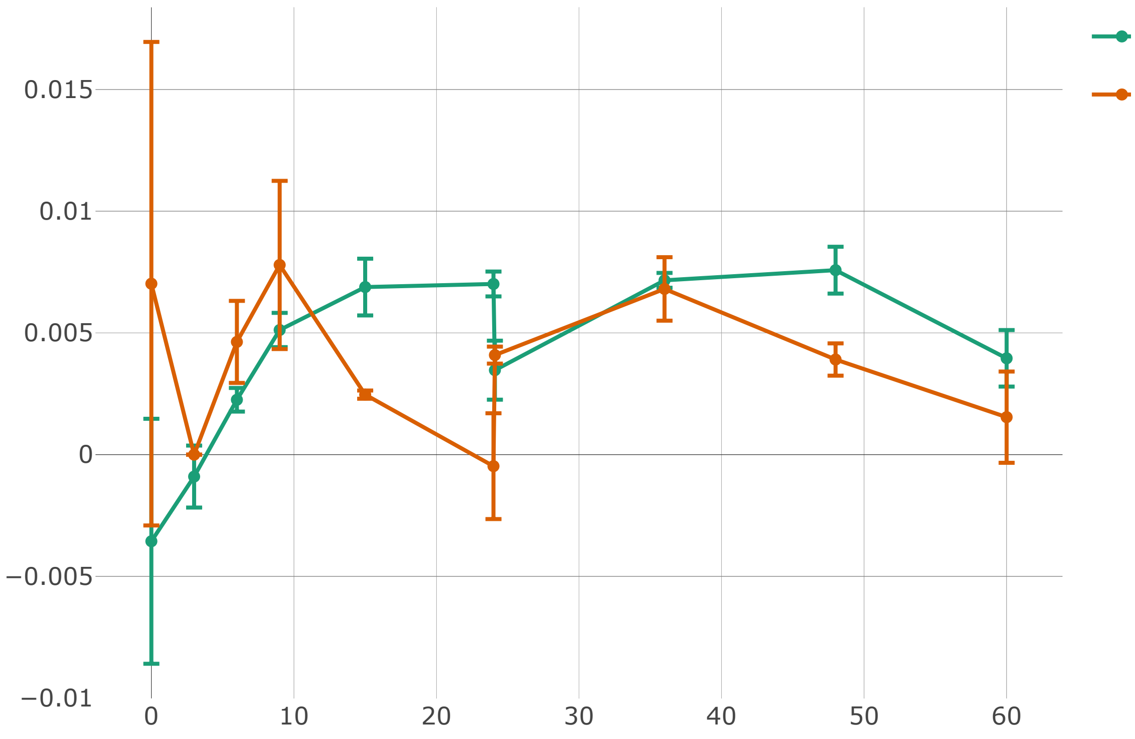

glc__D Specific Productivity vs time for different

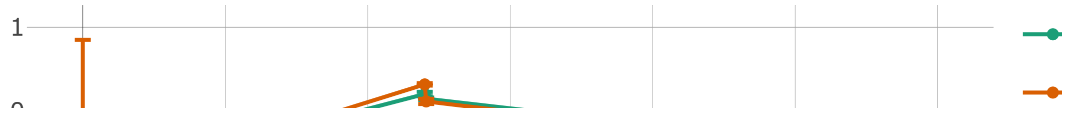

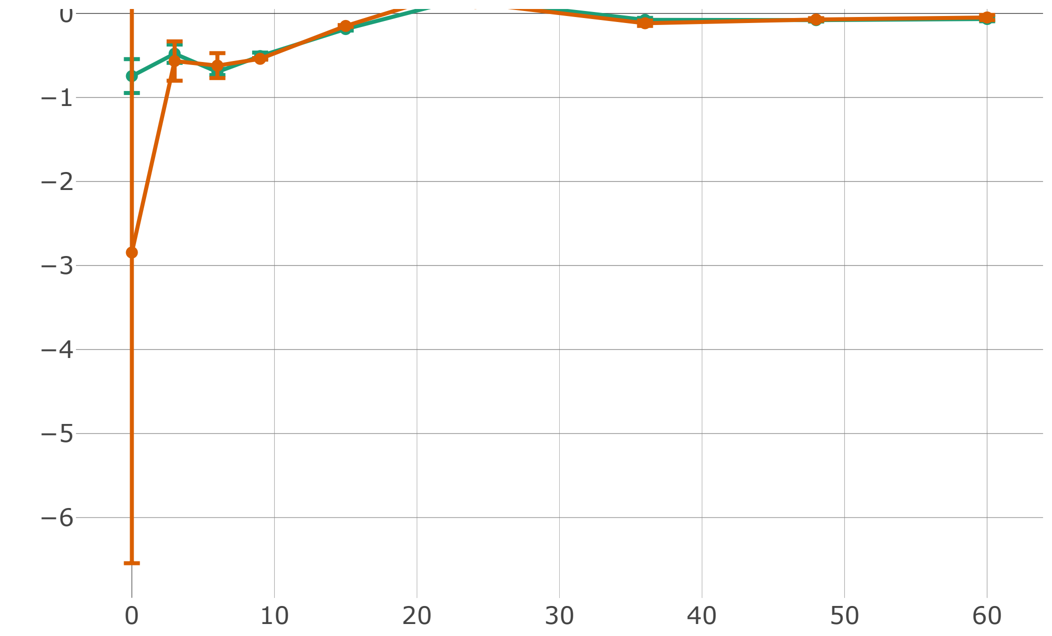

13bdo Specific Productivity vs time for different s

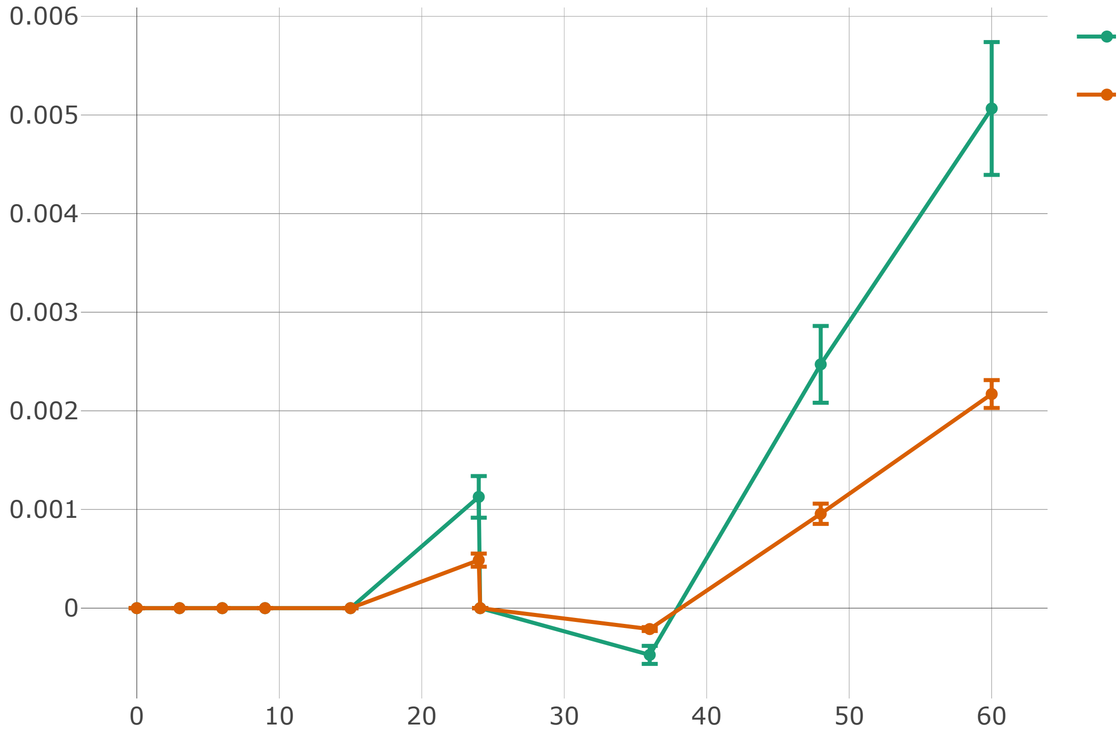

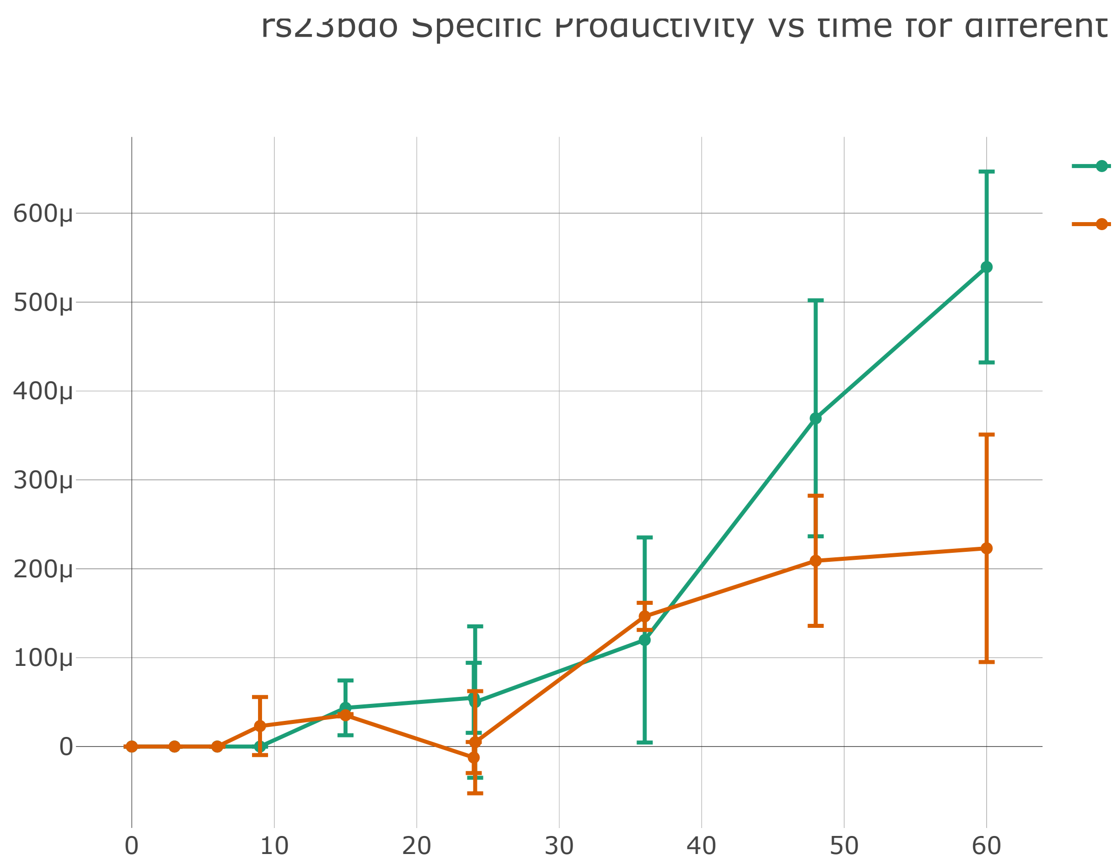

rs23bdo Specific Productivity vs time for different
600μ
500μ
400μ
300μ
200μ
100μ
0
0
10
20
30
40
50
60

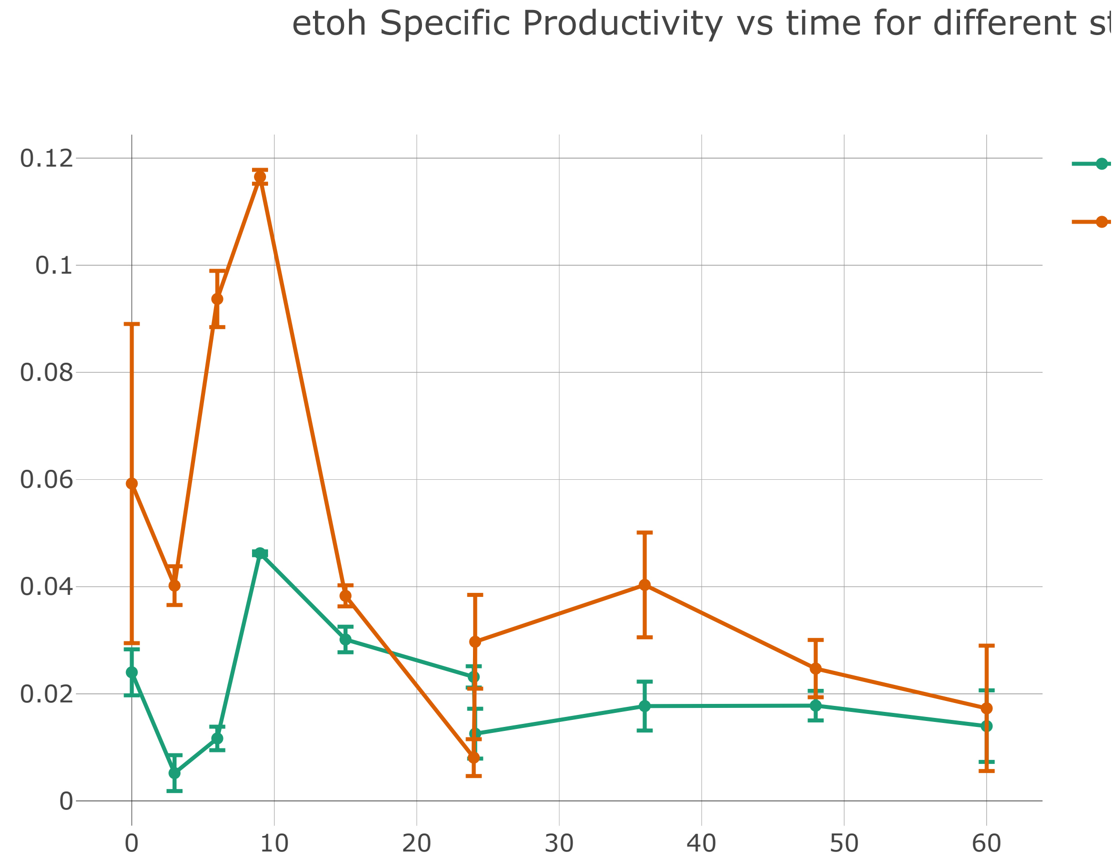

etoh Specific Productivity vs time for different st
0.12
0.1
0.08
0.06
0.04
0.02
0
0
10
20
30
40
50
60

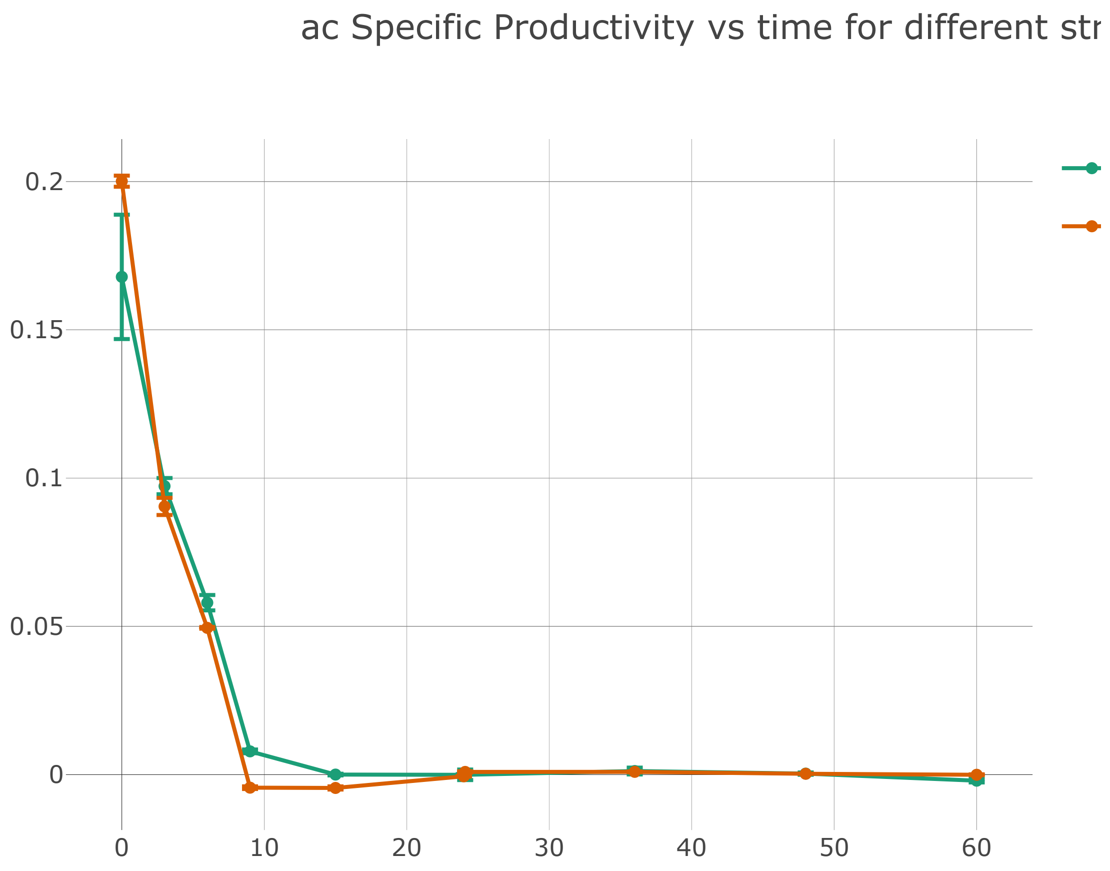
ac Specific Productivity vs time for different str
0.2
0.15
0.1
0.05
0
0
10
20
30
40
50
60

In [12]:

```
implot.plot_analyte_value_orderby_basemedia(expt)
```

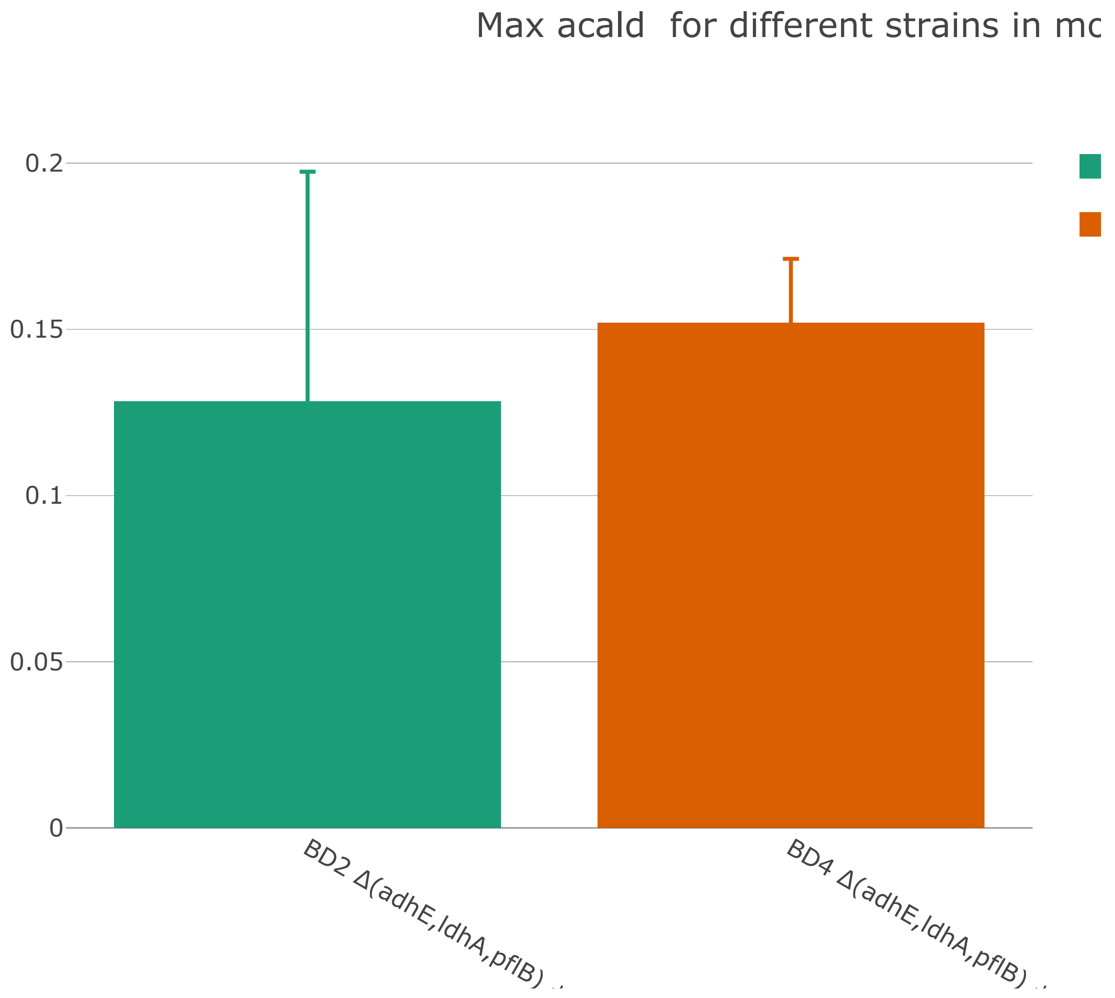

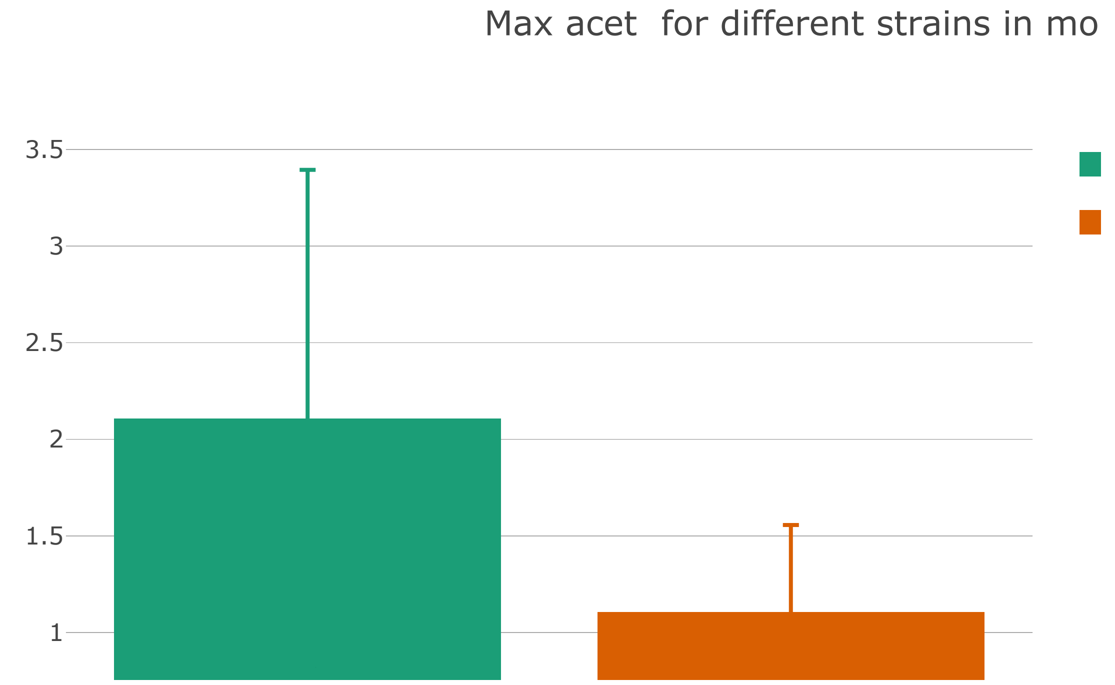

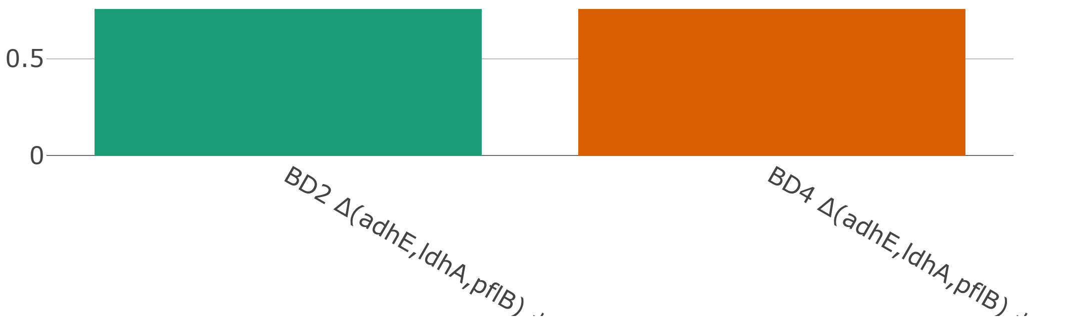
0.5
0
BD2 Δ(adhE,ldhA,pflB) ..
BD4 Δ(adhE,ldhA,pflB) ..

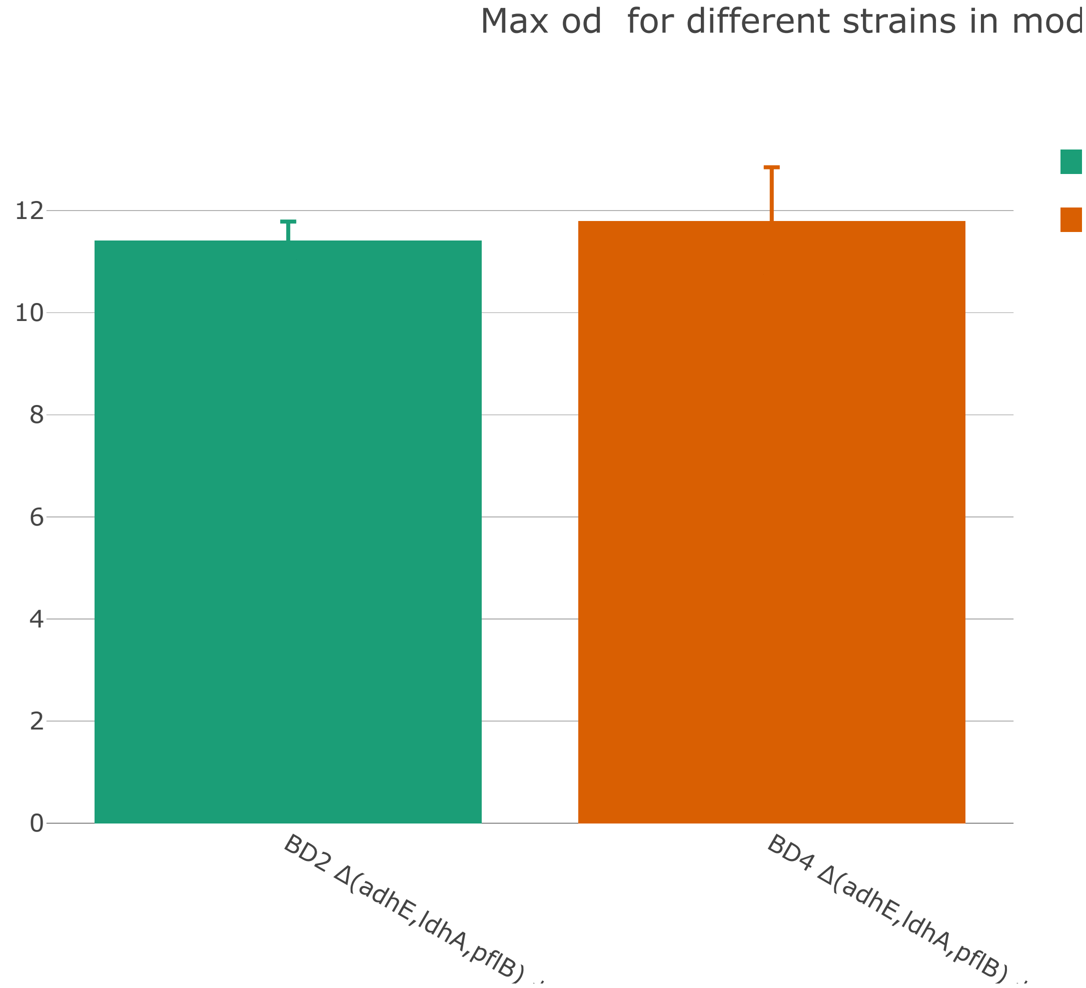
Max od for different strains in mod
12
10
8
6
4
2
0
BD2 Δ(adhE,ldhA,pflB) ..
BD4 Δ(adhE,ldhA,pflB) ..

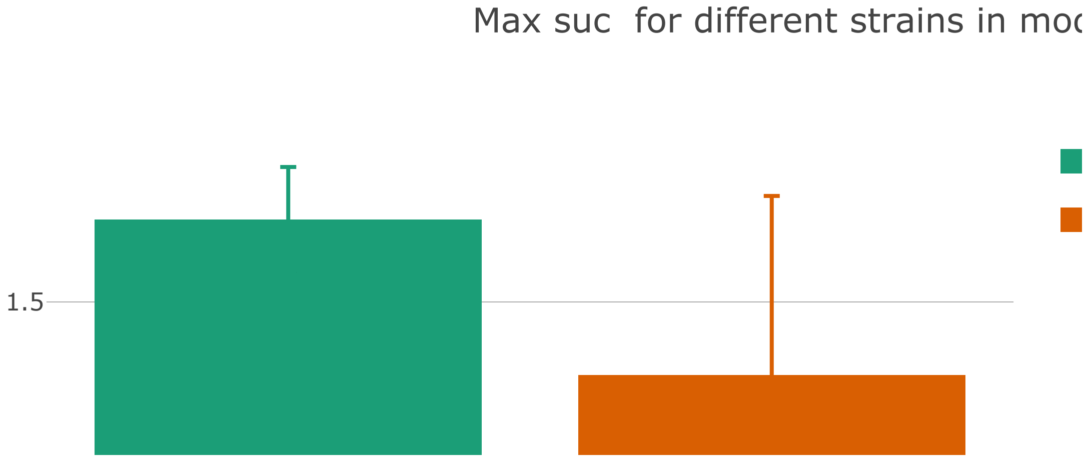
Max suc for different strains in moc
1.5

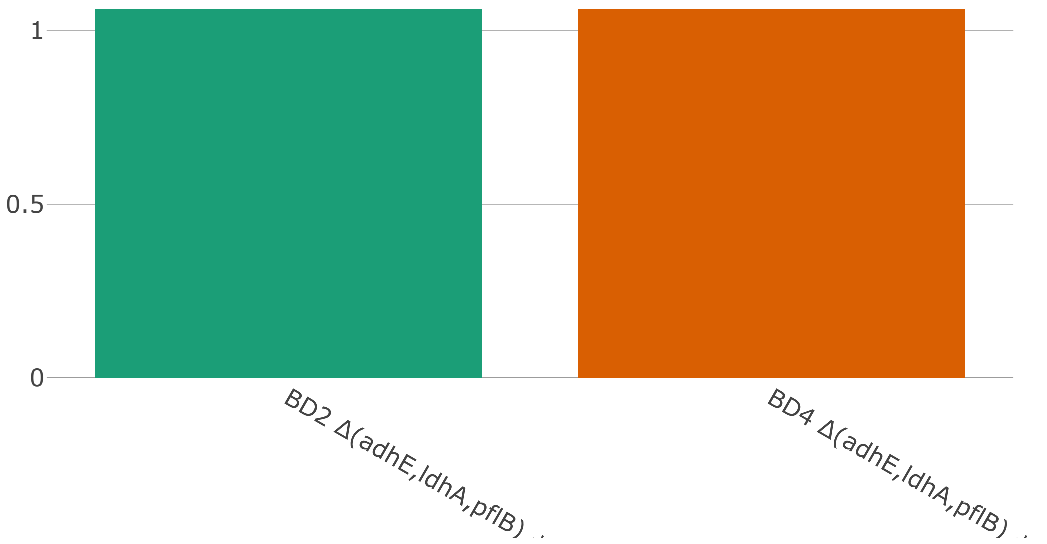

Max m23bdo for different strains in m

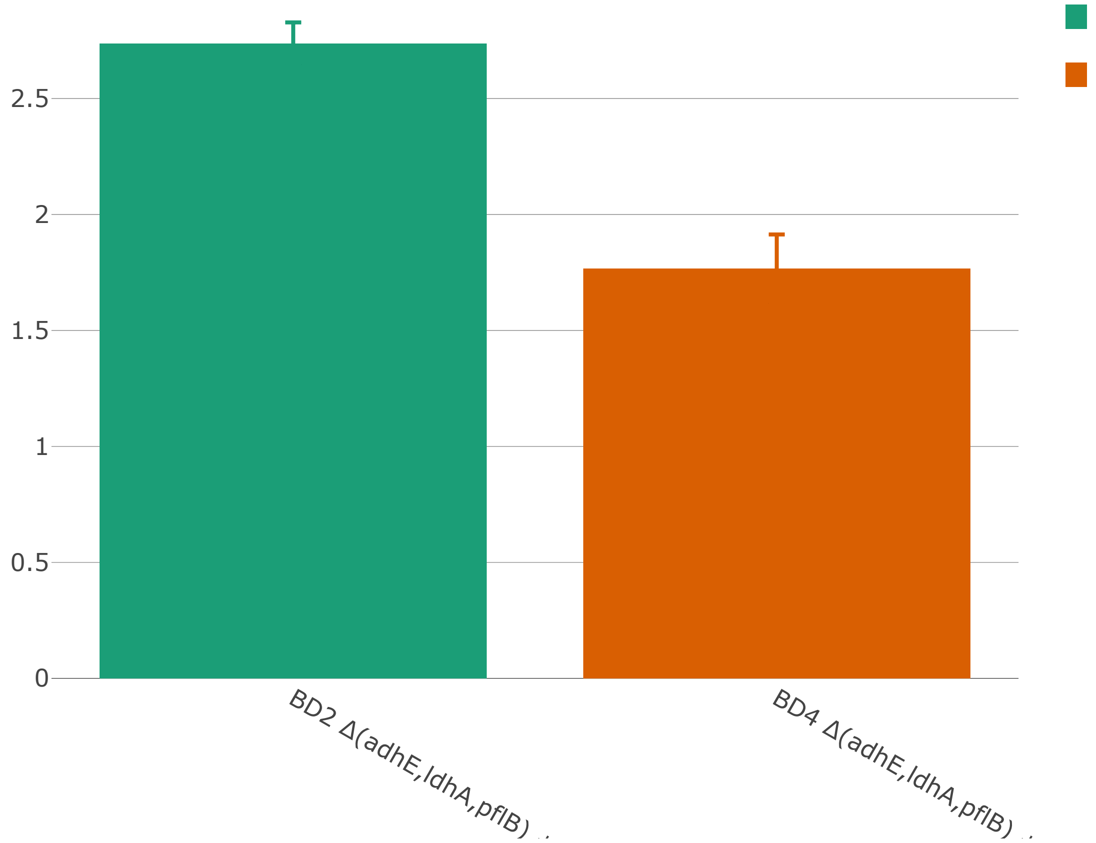

Max glc__D for different strains in m

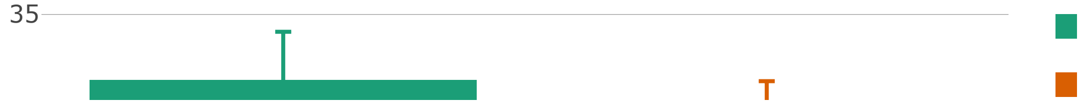

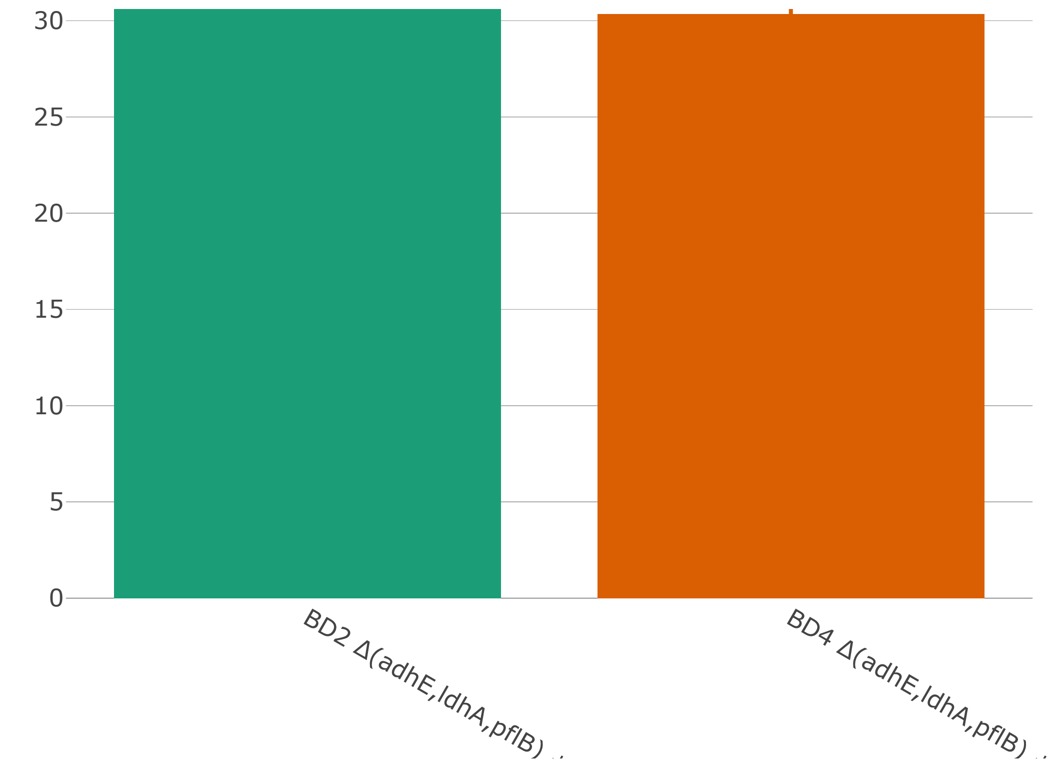

Max 13bdo for different strains in mo

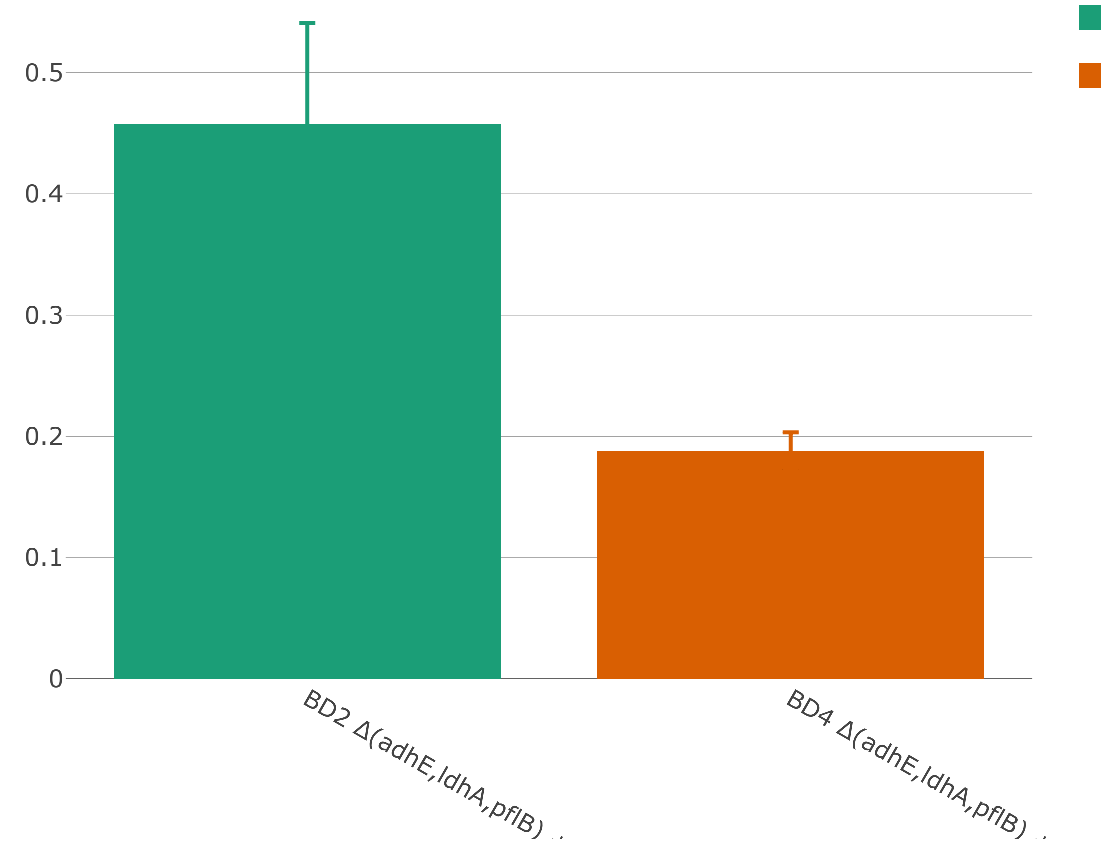

[illegible]

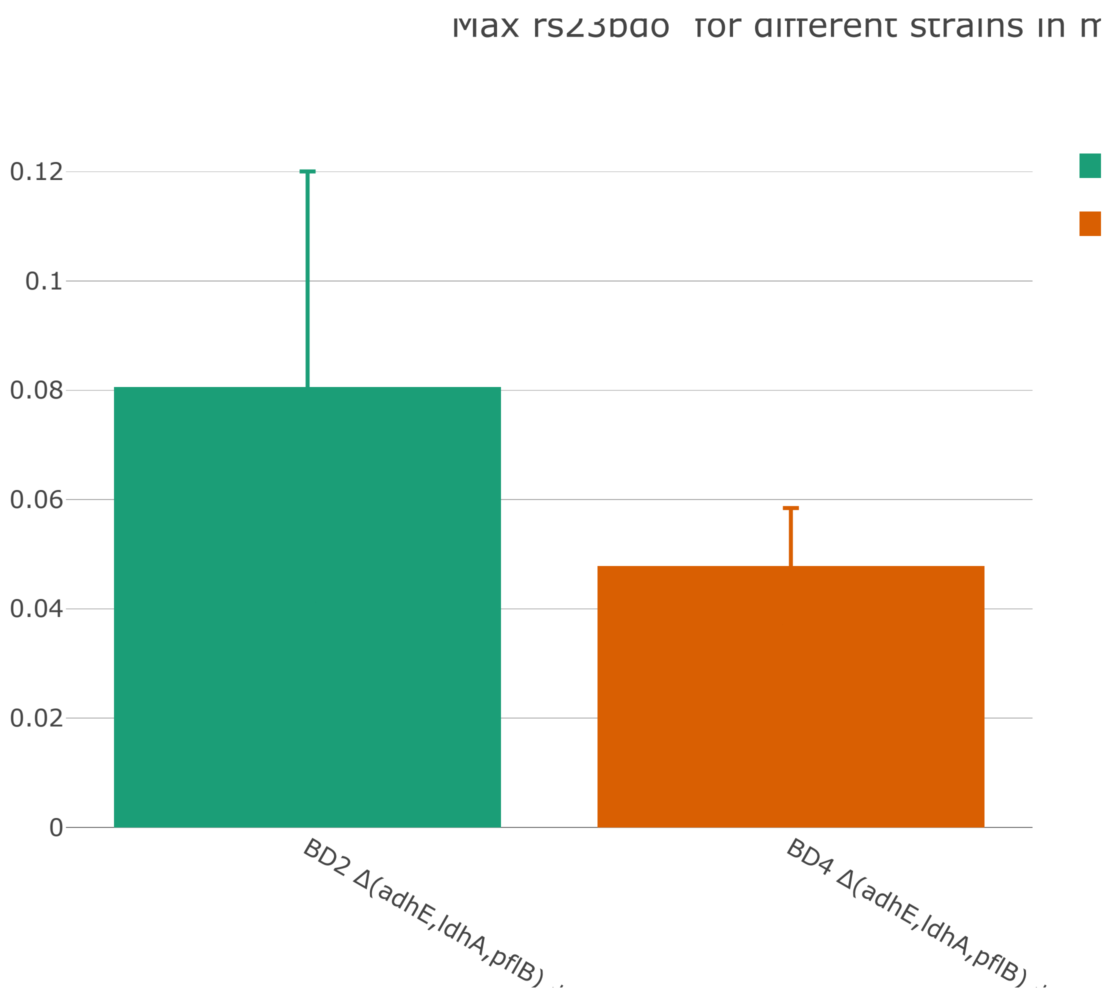
Max rs23bdo for different strains in m
0.12
0.1
0.08
0.06
0.04
0.02
0
BD2 Δ(adhE,ldhA,pflB)
BD4 Δ(adhE,ldhA,pflB)

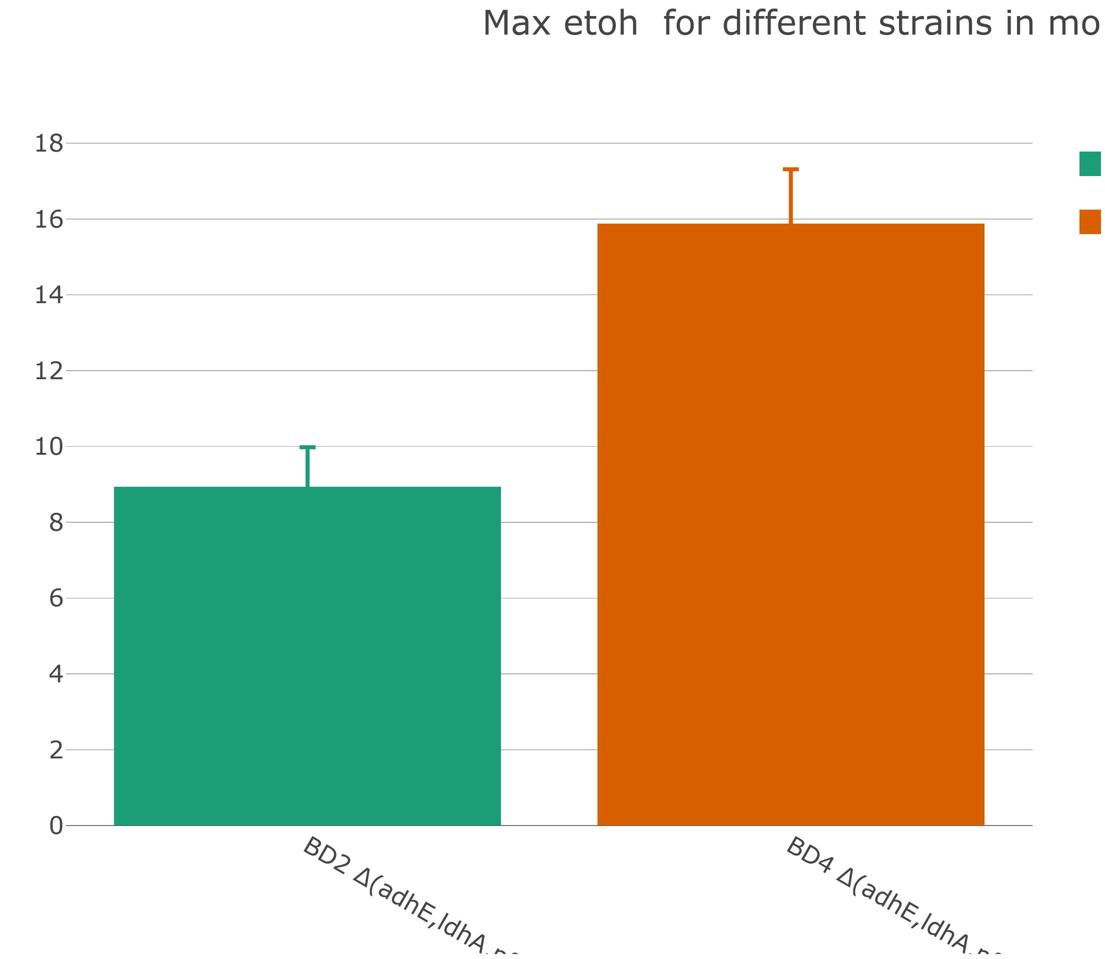
Max etoh for different strains in mo
18
16
14
12
10
8
6
4
2
0

,pflB) ..
,pflB) ..

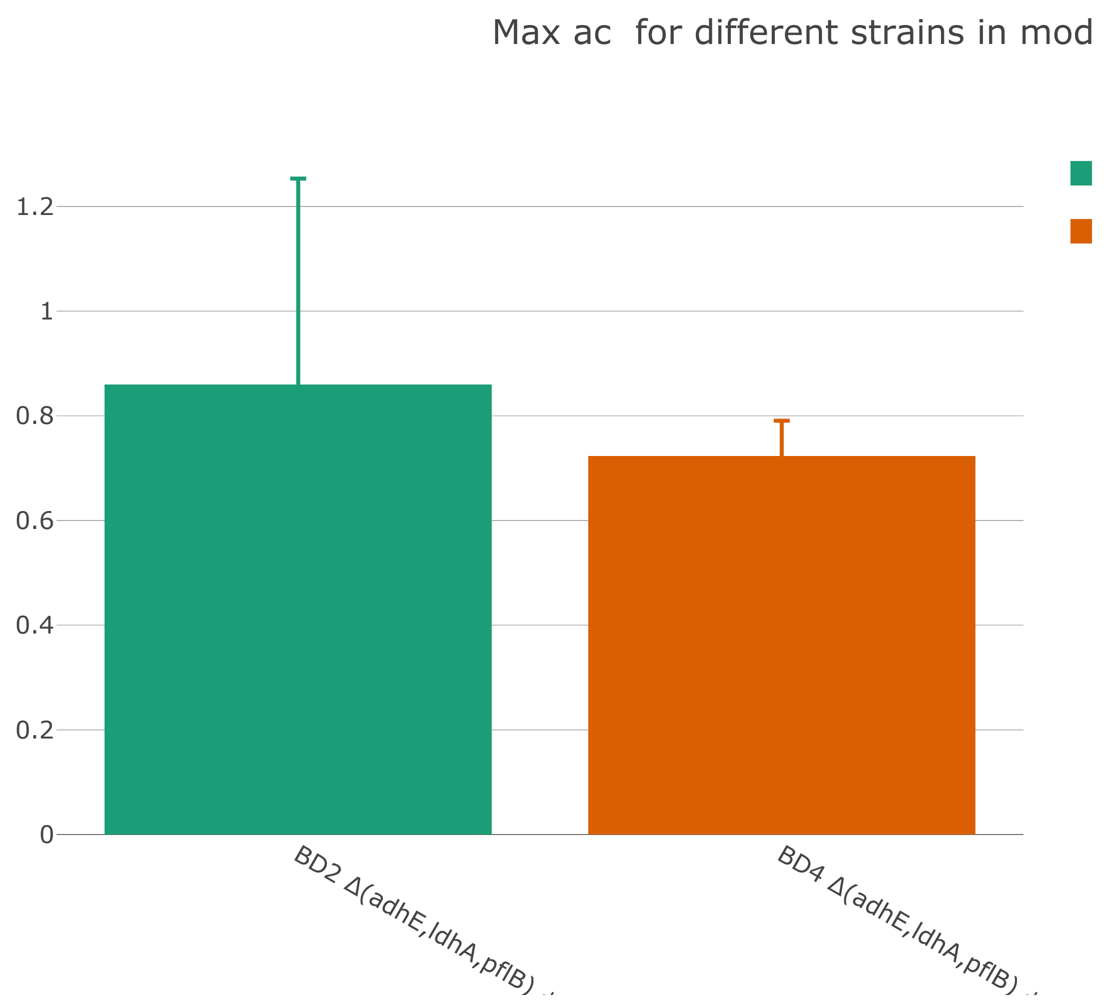
Max ac for different strains in mod
1.2
1
0.8
0.6
0.4
0.2
0
BD2 Δ(adhE,ldhA,pflB) ..
BD4 Δ(adhE,ldhA,pflB) ..

In [ ]:
