# Supplementary material for: Impact framework: A python package for writing data analysis workflows to interpret microbial physiology
Source: Metab Eng Commun. 2019 Apr 4;9:e00089. doi: 10.1016/j.mec.2019.e00089 (PMC6462781; doi:10.1016/j.mec.2019.e00089)
Supplement: Multimedia component 3 [file mmc3.pdf]

Supplementary information

Table **S1**: Default titers data format, typical of HPLC

	glucose substrate	pyruvate product	succinate product	OD600 biomass
strain1,plasmid1,,1,0	30.77	0	0	0.063904
strain1,plasmid1,,1,3	32.71	0	0	0.135195
strain1,plasmid1,,1,6	35.55	0	0	0.361593
strain1,plasmid1,,1,9	33.95	0	0.01	1.25
strain1,plasmid1,,1,12	29.16	0	0.01	3.10
strain1,plasmid1,,1,15	21.99	0	0.09	3.32
strain1,plasmid1,,1,24	9.57	0	1.20	9.73
strain1,plasmid1,,1,24.1	40.95	0	0.995	10.10

Table **S2**: SoftMax Pro OD data format

##BLOCKS= 1				
Plate:	Plate#1	1.3	PlateFormat	Kinetic
Time(hh:mm:ss)	Temperature(¡C)	1	2	3
0:00	37.00	0.1467	0.1375	0.148
		0.1269	0.1336	0.137
		0.1369	0.122	0.1536
		0.1509	0.1637	0.1592
		0.157	0.1484	0.1638
		0.1814	0.1928	0.1779
		0.0669	0.0623	0.0691
		0.0791	0.0998	0.0598
15:00	37.00	0.164	0.1594	0.1652
		0.1377	0.1462	0.1514
		0.1508	0.1255	0.1858
		0.1701	0.1831	0.1789

Table **S3**: Default kinetic data format, typical of plate readers.

	1	2	3	4
A	Identifier	0	900	1800
B	strain:MG|ko:pgi|media__base:LIMS|rep:1	0.1743	0.1923	0.1937
C	strain:MG|ko:pgi|media__base:LIMS|rep:2	0.1649	0.1773	0.1803
D	strain:MG|ko:pgi|media__base:LIMS|rep:3	0.1649	0.1819	0.1846
E	strain:MG|ko:pgi|media__base:LIMS|rep:4	0.1251	0.1332	0.1359
